# Value-related learning in the olfactory bulb occurs through pathway-dependent perisomatic inhibition of mitral cells

**DOI:** 10.1371/journal.pbio.3002536

**Published:** 2024-03-01

**Authors:** Sander Lindeman, Xiaochen Fu, Janine Kristin Reinert, Izumi Fukunaga

**Affiliations:** Sensory and Behavioural Neuroscience Unit, Okinawa Institute of Science and Technology Graduate University, Okinawa, Japan; Ecole Polytechnique Federale de Lausanne, SWITZERLAND

## Abstract

Associating values to environmental cues is a critical aspect of learning from experiences, allowing animals to predict and maximise future rewards. Value-related signals in the brain were once considered a property of higher sensory regions, but their wide distribution across many brain regions is increasingly recognised. Here, we investigate how reward-related signals begin to be incorporated, mechanistically, at the earliest stage of olfactory processing, namely, in the olfactory bulb. In head-fixed mice performing Go/No-Go discrimination of closely related olfactory mixtures, rewarded odours evoke widespread inhibition in one class of output neurons, that is, in mitral cells but not tufted cells. The temporal characteristics of this reward-related inhibition suggest it is odour-driven, but it is also context-dependent since it is absent during pseudo-conditioning and pharmacological silencing of the piriform cortex. Further, the reward-related modulation is present in the somata but not in the apical dendritic tuft of mitral cells, suggesting an involvement of circuit components located deep in the olfactory bulb. Depth-resolved imaging from granule cell dendritic gemmules suggests that granule cells that target mitral cells receive a reward-related extrinsic drive. Thus, our study supports the notion that value-related modulation of olfactory signals is a characteristic of olfactory processing in the primary olfactory area and narrows down the possible underlying mechanisms to deeper circuit components that contact mitral cells perisomatically.

## Abstract 2

Reward-driven learning begins in the very early stages of sensory processing. This study reinforces the idea that value-related modulation of olfactory signals is a key aspect of primary olfactory processing, and identifies potential mechanisms by characterizing deeper circuit components that interact with the soma of mitral cells.

## Introduction

Sensory systems of the brain play crucial roles in guiding animals’ choices. One such role played by the systems is in reward-driven learning, where the internal representations of sensory cues are adjusted as a result of past reward encounters, to influence their future behavioural choices. In addition to long-term adjustments, decades of studies across brain areas have demonstrated that reward expectations are potent and dynamic modulators of sensory activity. For example, stimulus evoked responses in many sensory regions of the brain scale with the quantity of expected reward [1–5], which is often interpreted as representations of the subjective value [6,7]. Such a system where sensory processing is fine-tuned flexibly may be crucial for maximising returns in a dynamic and uncertain world [7].

Decision and value-related modulations of sensory responses are featured prominently in higher sensory areas [1,8]. However, recent studies indicate that even early stages of sensory processing, especially in rodents, participate in value-like representations [9–11]. The olfactory system is an extreme case in this regard, where apparent reward-related modulation is readily observed as peripherally as in the olfactory bulb [12,13], the primary olfactory region situated just one synapse away from the site of sensory transduction. This peripheral location, along with the saliency of olfactory cues for rodents, makes the olfactory bulb an attractive structure to study the mechanisms that generate value-like signals in the brain [12].

The nature of this apparent reward-related modulation in the olfactory bulb remains unresolved. For example, one study observed that evoked responses to rewarded versus unrewarded odours in the olfactory bulb diverge only transiently during learning [12]. Such a transient modulation could be explained by dynamic changes in the level of animal’s engagement [14], where the learning-related modulation corresponds mainly to the changes in the inputs from the sensory periphery arising from sniff pattern changes. Rodents indeed adjust the odour sampling patterns exquisitely according to the behavioural contexts [15,16]. However, given that the olfactory bulb is a major target of feedback and neuromodulatory projections from many brain regions, value-related information could affect how the olfactory bulb represents odours. For example, electrical and optogenetic stimulations and pharmacological manipulations of neuromodulatory and feedback inputs to the olfactory bulb change the gain of odour responses in the principal neurons of this region [17–22].

In general, the effects of such perturbations depend on the output neuron type—mitral cells versus tufted cells. Mitral and tufted cells of the olfactory bulb differ physiologically and morphologically [23–27] and project to different downstream areas [26]. As a result, they are regarded as the starting points of parallel olfactory processing. Possible origins of the cell type-dependent modulation may include different sources of modulatory signals [17], receptor types expressed [28], or connectivity with distinct sets of local interneurons [25,29–31]. For example, a recent study in naïve mice demonstrated that feedback modulations of mitral versus tufted cells preferentially involve the piriform cortex versus the anterior olfactory cortex, respectively [17]. Within the olfactory bulb, it is yet unknown how diverse modulatory signals originating from different brain regions reach the output neurons in a cell type-specific manner. Resolving the nature and mechanisms underlying the cell type-specific modulation is crucial for understanding how long-range inputs from multiple brain regions couple into the intricate local circuitry to alter their computations.

Here, we show that the olfactory bulb exhibits robust and consistent reward-related signals during a trace olfactory conditioning paradigm, where mice discriminate between closely related olfactory mixtures. This phenomenon is characterised by widespread inhibitory responses following the rewarded odour presentation, in mitral cells but not tufted cells. This divergence is not explained by the odour identity or sampling strategy and reflects the congruence of sensory drive and contextual signals. By imaging from specific subcellular compartments of mitral cells, we demonstrate that the divergent responses first become evident perisomatically. Depth-resolved imaging from the dendrites of adult-born granule cells suggests that the cell type-specific modulation may involve an extrinsic drive to putative mitral cell-targeting granule cells.

## Results

**Fig 1:**
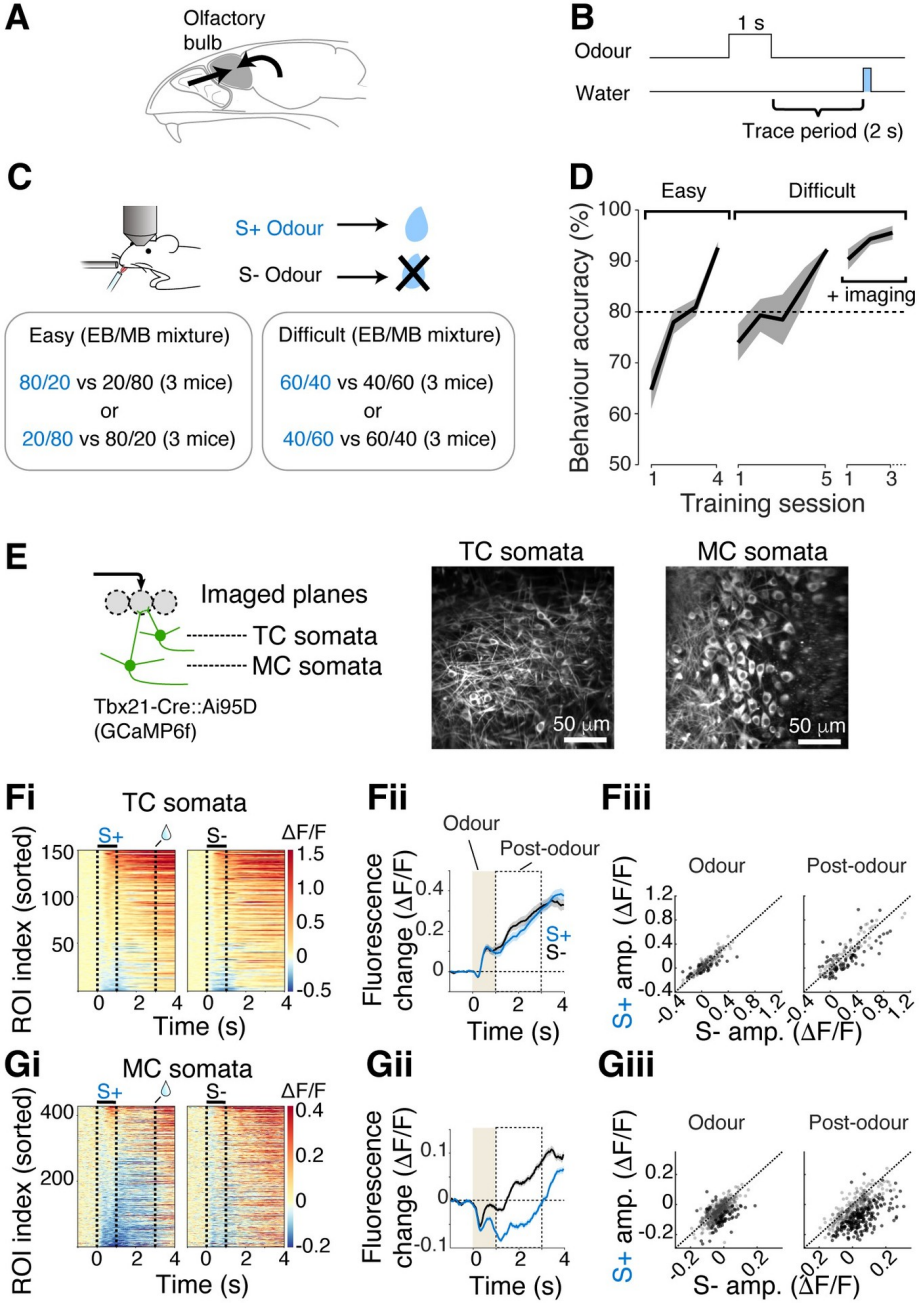
Widespread inhibition is observed in mitral cells in response to rewarded odour. (**A**) Schematic showing 2 major sources of inputs to the olfactory bulb. Left arrow represents olfactory nerve inputs. Right arrow represents long-range inputs from other brain areas. (**B**) Odour was presented for 1 s and did not overlap with the reward that was delivered 2 s after the odour offset. (**C**) Odours used in the Go/NoGo olfactory discrimination tasks. Blue font corresponds to the rewarded (S+) odour. Mice were head-fixed and had a cranial window implanted. (**D**) Time course of task acquisition for easy and difficult discriminations defined in **C**. Imaging sessions took place in proficient mice (*n* = 6 mice). (**E**) Left, imaging configuration. Mitral cell (MC) and tufted cell (TC) somata were distinguished by depth. Right, example fields of view for TC somata and MC somata. (**Fi–iii**) Responses to S+ and S- odours measured in TC somata. (**Fi**) Colormap representation of fluorescence change over time for all ROIs. (**Fii**) Average responses to S+ (blue) and S- (black) odours from all ROIs. (**Fiii**) Scatter plot comparing S- vs. S+ response amplitudes for the period shown in **Fii**. Each point represents 1 ROI, and the data shown are from all sessions and mice. Dotted line represents unity (S- amplitude = S+ amplitude). Individual points correspond to ROIs. Black dots indicate S+ and S- responses that were significantly different. (**Gi–iii**) Same as **Fi–iii**, but for MC somata. *N* = 150 and 428 ROIs, and 3 and 6 mice for TC somata and MC somata, respectively. Source data can be found in Fig 1 data, Dryad.

The olfactory bulb integrates both feedforward sensory stimuli, as well as long-range projections from other brain areas (**Fig 1A**). The latter input is thought to convey behavioural contextual signals to the olfactory bulb and tune activity patterns flexibly. To study how the behavioural context modulates the olfactory bulb output in olfactory decision-making, we trained head-fixed mice to perform an olfactory discrimination task (**Fig 1**). Water-restricted mice were trained to associate a rewarded odour (S+ odour) with a water reward, and an unrewarded odour (S- odour) with no water delivery (**Fig 1B**). Note that this paradigm includes a trace period, as we reasoned that an early cessation in the feedforward signal may maximise the chance of observing context-related activity patterns.

The mice were first trained to discriminate between easily distinguishable odour mixtures, which comprised ethyl butyrate (EB) and methyl butyrate (MB), mixed at 80%/20% ratio versus a 20%/80% ratio for the S+ versus S- odours, respectively (**Fig 1C**). When the mice reached a criterion of 80% accuracy (3 ± 0.9 days, *n* = 6 mice, **Fig 1D**), they were trained to discriminate between more similar odour mixtures (“Difficult discrimination task”; 60%/40% mixture of EB and MB versus a 40%/60% mixture). This is a task known to engage many components of the olfactory bulb circuitry [32]. Well-trained mice discriminated between these similar mixtures in 1.63 ± 0.53 s (**S1 Fig**), with comparable sniffing patterns for the S+ versus S- odours (**S2 Fig**), consistent with previous reports where similar odours and reward timing were used [33,34].

In mice proficiently performing the difficult olfactory discrimination task, we studied the responses of olfactory bulb outputs to the S+ versus S- odours. The calcium indicator GCaMP6f was expressed in mitral and tufted cells using Tbx21-Cre mice crossed with Ai95D mice [35,36] and was imaged using a two-photon microscope (*n* = 428 regions of interest (ROIs) in 6 mice, and *n* = 150 ROIs in 3 mice, respectively; **Figs 1E–1G, and S3**). Mitral and tufted cells were distinguished by depth (**Fig 1E**). Tufted cells responded largely similarly to both odours (mean ΔF/F during odour = 0.628 ± 0.135 and 0.655 ± 0.148 for S+ and S-, respectively; *p* = 0.777, Wilcoxon rank-sum test; mean ΔF/F post-odour = 0.203 ± 0.264 and 0.237 ± 0.264 for S+ and S-, respectively; *p* = 0.149, Wilcoxon rank-sum test; **Fig 1F**). Peculiarly, responses of the mitral cell somata to the S+ odour were characterised by widespread inhibitory responses (mean ΔF/F S+ = −0.048 ± 0.058; S- = −0.022 ± 0.054; *p* < 0.001, Wilcoxon rank-sum test; **Fig 1G**). We also observed that the S+ odour evoked less inhibition on trials where mice did not generate anticipatory licks (**S4 Fig**). This dominance of inhibition for the S+ odour was present soon after the odour onset but was particularly pronounced during the post-odour period (mean ΔF/F S+ = −0.048 ± 0.095; S- = 0.034 ± 0.102; *p* < 0.001, Wilcoxon rank-sum test; **Fig 1G**). The earliest time when the S+ and S- responses diverge significantly was 818 ± 540 ms after the odour onset (mean ± standard deviation; *n* = 16 fields of view, 6 mice).

**Fig 2:**
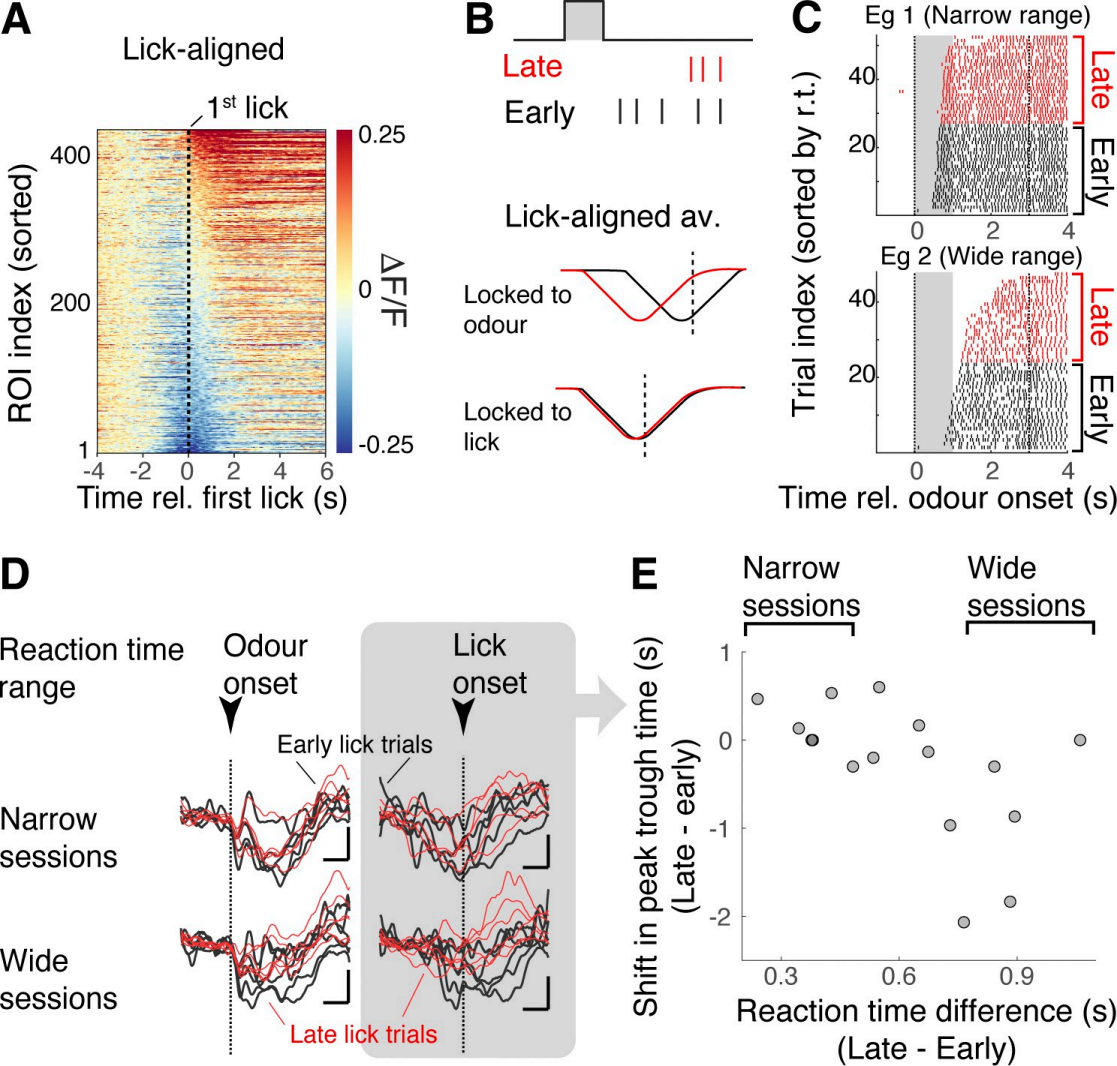
Reward-related inhibition is locked to the odour presentation. (**A**) Fluorescence change from mitral cell somata aligned to the time of first anticipatory lick after odour onset (S+ trials). (**B**) Predictions for 2 alternative hypotheses for late-lick vs. early-lick trials; if inhibition is generally odour-locked, a shift in the peak inhibition is observed in the lick-aligned average. If the reward-related inhibition is locked to generation of licks, the trough times will be the same for late vs. early lick trials relative to the onset time of anticipatory lick. (**C**) Lick raster plots from 2 example sessions. Late (early) vs. early (black) lick trials were defined as trials where the first anticipatory lick occurred later or earlier than the median lick onset time for each session. The second dotted line represents the timing of water delivery. (**D**) Lick-aligned averages for early vs. late lick for all sessions (black and red traces, respectively). Each trace is an average for 1 session. Top row is for 5 sessions with the smallest range in the reaction times (see panel **E**). Bottom row is for sessions where reaction times ranged more widely. (**E**) Shift in the peak trough time in the odour-aligned (left) and lick-aligned (right) averages compared against mean difference in the lick onsets for the late vs. early trials. Each point corresponds to 1 imaging session. Pearson’s correlation coefficient = −0.555, *p* = 0.026 (*n* = 16 sessions, 6 mice). Source data can be found in Fig 2 data, Dryad.

The late onset of the reward-associated inhibition in mitral cells raises the question regarding the underlying drive: Is the inhibitory component locked to the anticipatory motor output, or to the odour? To analyse this, we divided the rewarded trials into 2 sets based on the animals’ reaction times (“early onset” versus “late onset”) and reverse-correlated the GCaMP6f signals to the onsets of anticipatory signals (“lick-aligned average”; **Fig 2**). If the peak of inhibition in the averages occur at the same time for the early lick sets and late lick sets, it would imply that the majority of the inhibition is locked more to the behavioural output (**Fig 2B**). This analysis revealed, in contrast, that the time of peak inhibition is shifted depending on the reaction time (Pearson’s correlation coefficient = −0.555, *p* = 0.026; *n* = 16 fields of view, 6 mice; **Fig 2C–2E**), indicating that the inhibition is, on average, locked to the odour.

**Fig 3:**
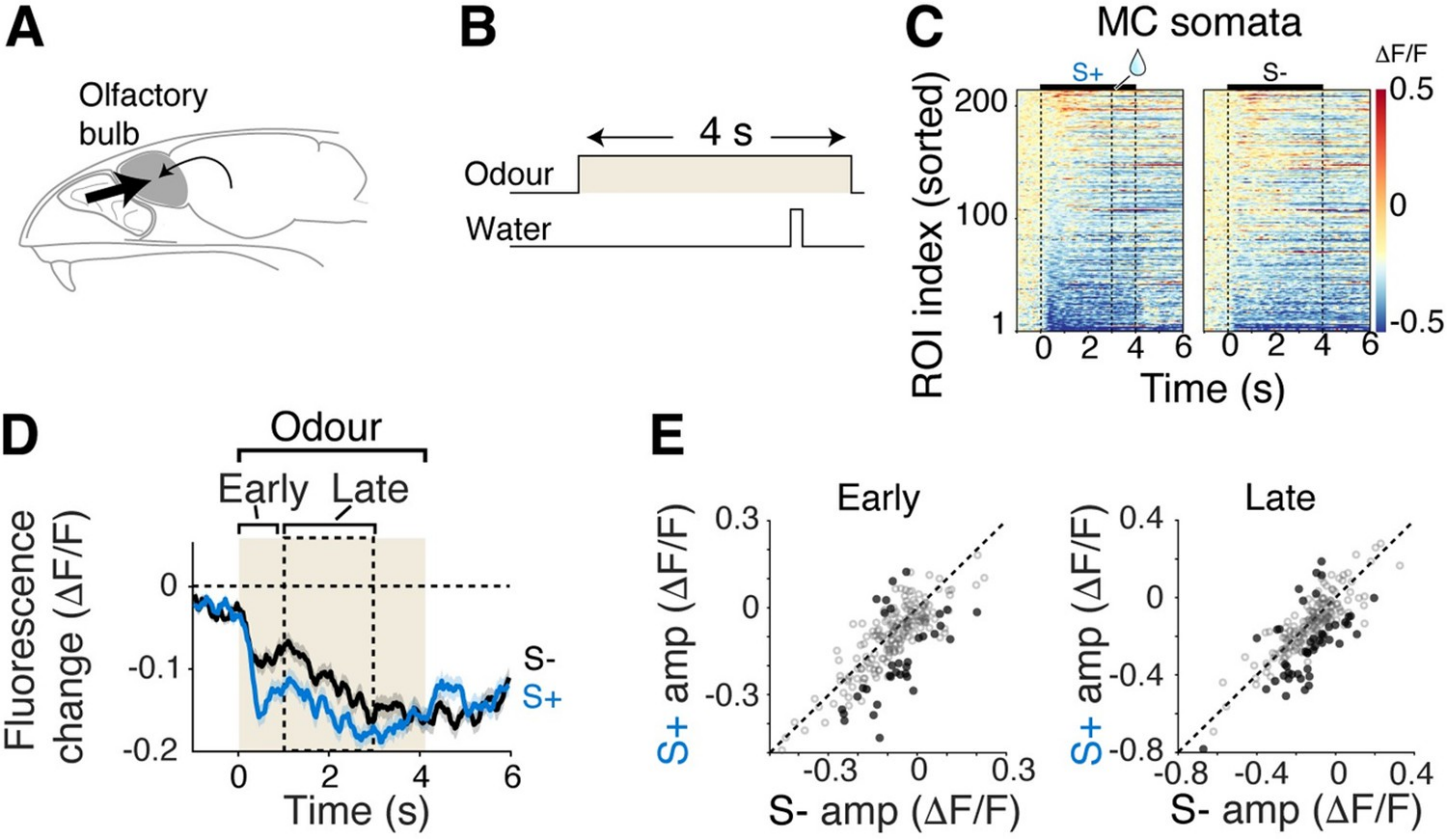
Longer odour presentation masks the appearance of divergent responses. (**A**) Schematic showing dominance of sensory drive. (**B**) A 4-s odour pulse overlapped temporally with reward delivery. (**C**) Colour map display showing GCaMP6f fluorescence change from mitral cell somata evoked by S+ and S- odours. (**D**) Average fluorescence change from all ROIs in response to S+ (blue) and S- (black) odours. Mean and SEM shown. (**E**) Scatter plots of average fluorescence change for S+ vs. S- odours for the time period indicated in **D**. Black dots indicate S+ and S- significantly divergent responses (*N* = 210 ROIs, 5 mice). Source data can be found in Fig 3 data, Dryad.

The prevalence of inhibitory responses in mitral cells following the rewarded odour presentation is striking, but this level of inhibitory dominance has not been reported previously, even though several studies already studied how mitral cells respond to odours during difficult odour discrimination paradigms [37–39]. The difference here may be the short duration of odour pulse used, followed by a 2-s long trace period. It is possible that, with a longer odour presentation, the feed-forward component may dominate over any intrinsic or modulatory influences in the olfactory bulb that underly the reward-related inhibition (**Fig 3A**). To test this possibility, in well-trained mice, we presented the odours for a longer period (4 s), making the task a delay task (**Fig 3B**). In this condition, mitral cells responded to the rewarded and unrewarded odours similarly (**Fig 3C–3E**). Notably, both responses were characterised by widespread inhibitory component (% of ROIs showing significant inhibition = 18.1 for S+ and 11.9 for S-, and 35.2 for S+ and 23.9 for S- in early and late time windows, respectively, *n* = 5 mice). While the divergent response is still present, the magnitude of this divergence is significantly reduced during long odour presentation (**S5 Fig**). This result indicates that the response divergence in the post-odour period may be uncovered when olfactory responses are allowed to evolve in the absence of feed-forward inputs.

**Fig 4:**
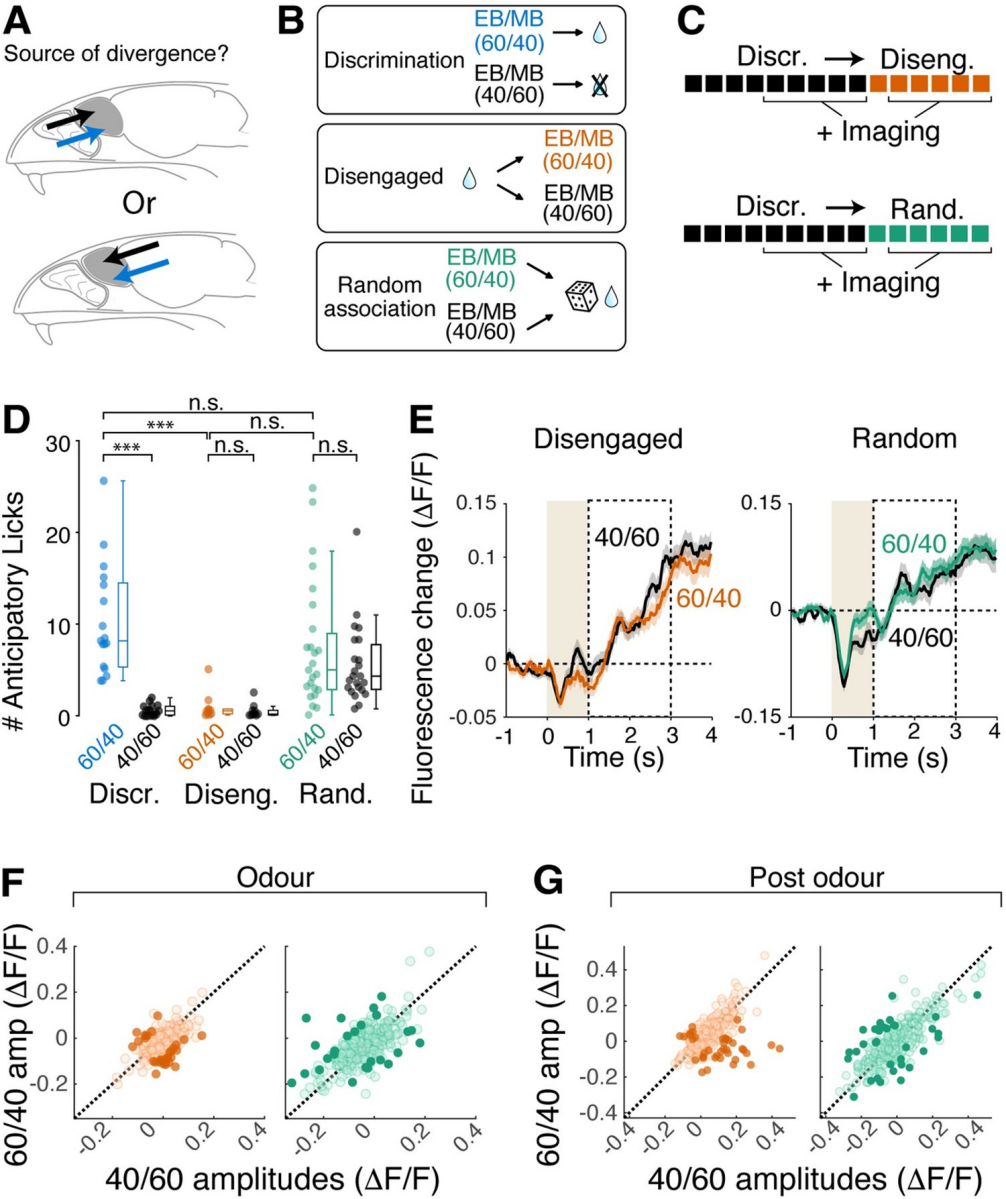
Occurrence of reward-related inhibition depends on the behavioural context, not odour identity. (**A**) Hypotheses on the source of signals underlying differential S+ and S- responses in mitral cell somata; it could derive from sensory stimuli (top) or from long-range inputs to the olfactory bulb (bottom). (**B**) Behavioural paradigms to decouple reward association while disengaging mice (middle) or engaging mice (“Random association”). In disengagement sessions, reward was delivered every trial, preceding odour presentations. In random association sessions, reward followed both mixtures of EB and MB 50% of the time. (**C**) Timeline of experiments. Mice first performed difficult olfactory discrimination, then went through either disengagement or random association sessions. Imaging took place from day 2 in both cases. (**D**) Number of anticipatory licks (licks within a 3-s window from odour onset) for the 2 odours for 3 behavioural paradigms. Individual points correspond to each imaging session analysed. *** Corresponds to *p* < 0.001 (post hoc Tukey–Kramer multiple comparisons after 1-way ANOVA). (**E**) Average fluorescence change of all ROIs (mitral cell somata) for the odours indicated. (**F**) Comparison of fluorescence change in response to the 2 odours for the odour period for disengagement sessions (left) and random association sessions (right). Individual points correspond to ROIs. Darker points represent significantly divergent responses. (**G**) Same as F, but for post-odour period. *N* = 125 ROIs, 3 mice for disengagement and 301 ROIs, 5 mice for random association. Source data can be found in Fig 4 data, Dryad.

To test if the behavioural state of the animal is crucial for the response divergence in mitral cells, we used 2 pseudo-conditioning paradigms using the same odours (**Fig 4A and 4B**). In the first case (“Disengaged”), the water was delivered every trial, approximately 15 s before the odour presentation (**Fig 4B**). In the second case (“Random association”), we delivered the water on randomly selected trials, so that both 60/40 and 40/60 odour mixtures were followed by water 50% of the time (**Fig 4B**). These 2 paradigms decouple the odour-reward association, while inducing different levels of engagement in the head-fixed mice [40]. Imaging sessions took place after the mice, previously trained on the difficult discrimination task, were switched to, and experienced at least 1 session of the new paradigm (**Fig 4C**).

In both control paradigms, the head-fixed mice showed no preferential licking for the 60/40 mixture (average anticipatory licks for disengagement paradigm = 1.5 ± 1.6 and 0.7 ± 0.8 on 60/40 and 40/60,respectively; *p* = 0.999, 1-way ANOVA with post hoc Tukey–Kramer multiple-comparisons; average anticipatory licks for random association paradigm = 6.9 ± 6.1 and 5.5 ± 4.3 on 60/40 and 40/60, respectively; *p* = 0.890, 1-way ANOVA with post hoc Tukey–Kramer multiple comparisons, **Fig 4D**). Importantly, disengagement and randomised paradigms differed in the general levels of anticipatory licks (average anticipatory licks for all trials = 1.1 ± 1.3 and 6.2 ± 5.2 for disengagement and random association paradigms, respectively; *p* = 0.0021, 1-way ANOVA with post hoc Tukey–Kramer multiple comparisons), indicating that different levels of behavioural engagement were indeed achieved by these paradigms. A difference between the 2 behavioural states included a general reduction in the mitral cell inhibition when the mice were disengaged, which is consistent with a previous observation [41]. Importantly, in both cases, the mitral cell somata responded similarly to the 2 odour mixtures (mean ΔF/F for disengagement = −0.02 ± 0.05 and −0.01 ± 0.06 during odour for 60/40 and 40/60, respectively; *p* = 0.465; for post-odour = 0.05 ± 0.09 and 0.05 ± 0.09; *p* = 0.553; mean ΔF/F for random association = −0.03 ± 0.09 and −0.04 ± 0.10 during odour; *p* = 0.259; post-odour = −0.01 ± 0.14 and 7.7 × 10^−5^ ± 0.14; *p* = 0.617, Wilcoxon rank-sum test; **Fig 4E–4G**). Note that the inhibition during the post-odour, anticipatory period that is normally present in discriminating mice was generally reduced in the 2 control paradigms. Curiously, the reward-related inhibition was reduced in mice performing an easy olfactory discrimination (**S6 Fig**). Together, these results indicate that the observed divergent responses in mitral cell somata are state dependent, and not explained by the odour identities.

What is the origin of the widespread inhibition associated with the rewarded odour? Previous studies showed that a variety of feedback and neuromodulatory projections to the olfactory bulb modulate the physiology of olfactory bulb neurons [18–20,42–44]. Further, several studies showed that such modulations manifest differently for mitral cells and tufted cells [17,18,38,45]. Recent works indicate that mitral cells receive more potent feedback modulation from the piriform cortex [17,18]. Further, anterior piriform cortex has been reported to contain neurons that show value-like signals [46]. Thus, even though it is beyond the scope of the current work to systematically investigate all sources, the anterior piriform cortex is a reasonable candidate for the source of the contextual signal resulting in the mitral cell-specific, reward-related inhibition we observe.

**Fig 5:**
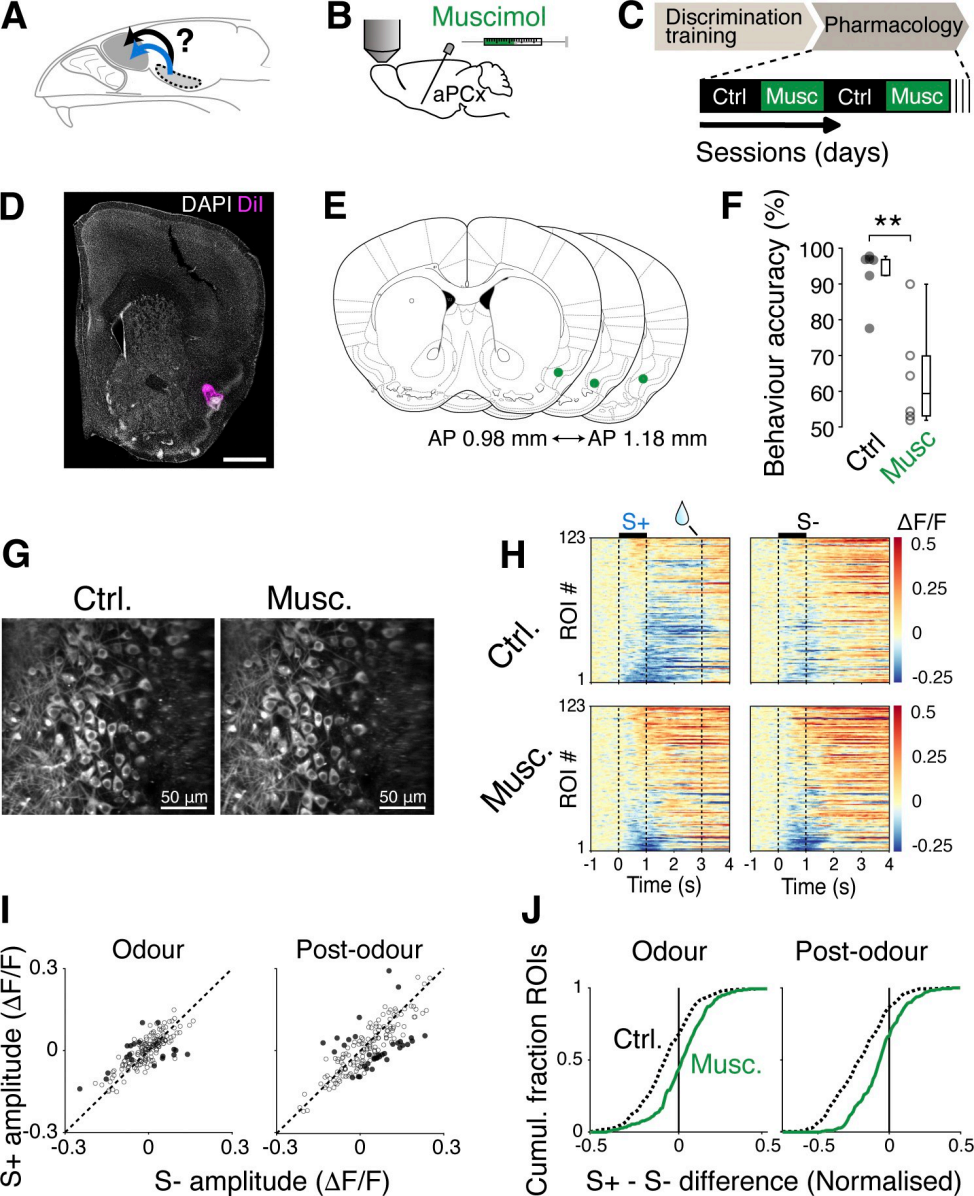
Intact piriform cortex is needed to observe the reward-related inhibition. (**A**) Hypothesis tested; anterior piriform cortex is necessary for mitral cell divergence during odour discrimination. (**B**) Muscimol solution (2 mM; 500 nL) was infused via an implanted cannula targeted to anterior piriform cortex. (**C**) Timeline of experiments. After mice were trained on the discrimination task, control and muscimol sessions alternated. One imaging session occurred per day. (**D**) Example of DiI location infused via an implanted cannula. Scale bar = 1 mm. (**E**) Summary of cannula tip locations. (**F**) Accuracy in performance (% of trials with correct lick response) for control vs. muscimol sessions. Individual points correspond to each imaging session. *P* = 0.004. (**G**) Example fields of view matched across 2 conditions. (**H**) Time course of fluorescence change for control (top) and muscimol (bottom) sessions in matched ROIs. (**I**) Average fluorescence change for each ROI for S+ and S- odours during odour and post-odour periods. Darker points represent significantly divergent responses. (**J**) Cumulative fraction of ROIs for normalised difference in fluorescence changes evoked by S+ and S-. *N* = 123 ROIs, 3 mice. Source data can be found in Fig 5 data, Dryad.

To test the involvement of the piriform cortex, we pharmacologically inactivated the ipsilateral anterior piriform cortex while the head-fixed mice performed the difficult olfactory discrimination task (**Fig 5A**). This was achieved by infusing the GABA_A_ agonist, muscimol, unilaterally through an implanted canula. Muscimol and control sessions were carried out on alternate days, but the same fields of view were sampled for the 2 conditions, so that the responses of the same ROIs could be compared directly. The infusion of muscimol disrupted the behavioural performance significantly (behavioural accuracy = 64.0 ± 14.5% during muscimol sessions; 92.8 ± 7.8% during control sessions; *p* = 0.004, Wilcoxon rank-sum test; *n* = 6 control sessions and 6 muscimol sessions, 3 mice; **Fig 5F**). When responses of mitral cells were imaged in this condition, divergence in the rewarded versus unrewarded odour responses was significantly reduced (mean ΔF/F during odour = −0.007 ± 0.073 and −0.023 ± 0.091 for S+ and S-, respectively; *p* = 0.174, Wilcoxon rank-sum test; mean ΔF/F post-odour = 0.067 ± 0.124 and 0.079 ± 0.171 for S+ and S-, respectively; *p* = 0.252, Wilcoxon rank-sum test, **Fig 5G–5I**). This was characterised by a reduction in the inhibitory responses evoked by the rewarded stimulus (normalised S+—S- difference = −0.007 ± 0.156 and 0.043 ± 0.142 in control and muscimol sessions respectively; *p* = 0.008, Wilcoxon rank-sum test; **Fig 5J**), and during the post-odour phase (normalised S+—S- difference = −0.263 ± 0.175 and −0.040 ± 0.168 in control and muscimol sessions, respectively; *p* = 3.53 × 10^−18^, Wilcoxon rank-sum test; **Fig 5J**). Together, these data indicate that an intact piriform cortex and/or accurate behavioural performance are required to observe the widespread inhibitory responses associated with the rewarded odour.

**Fig 6:**
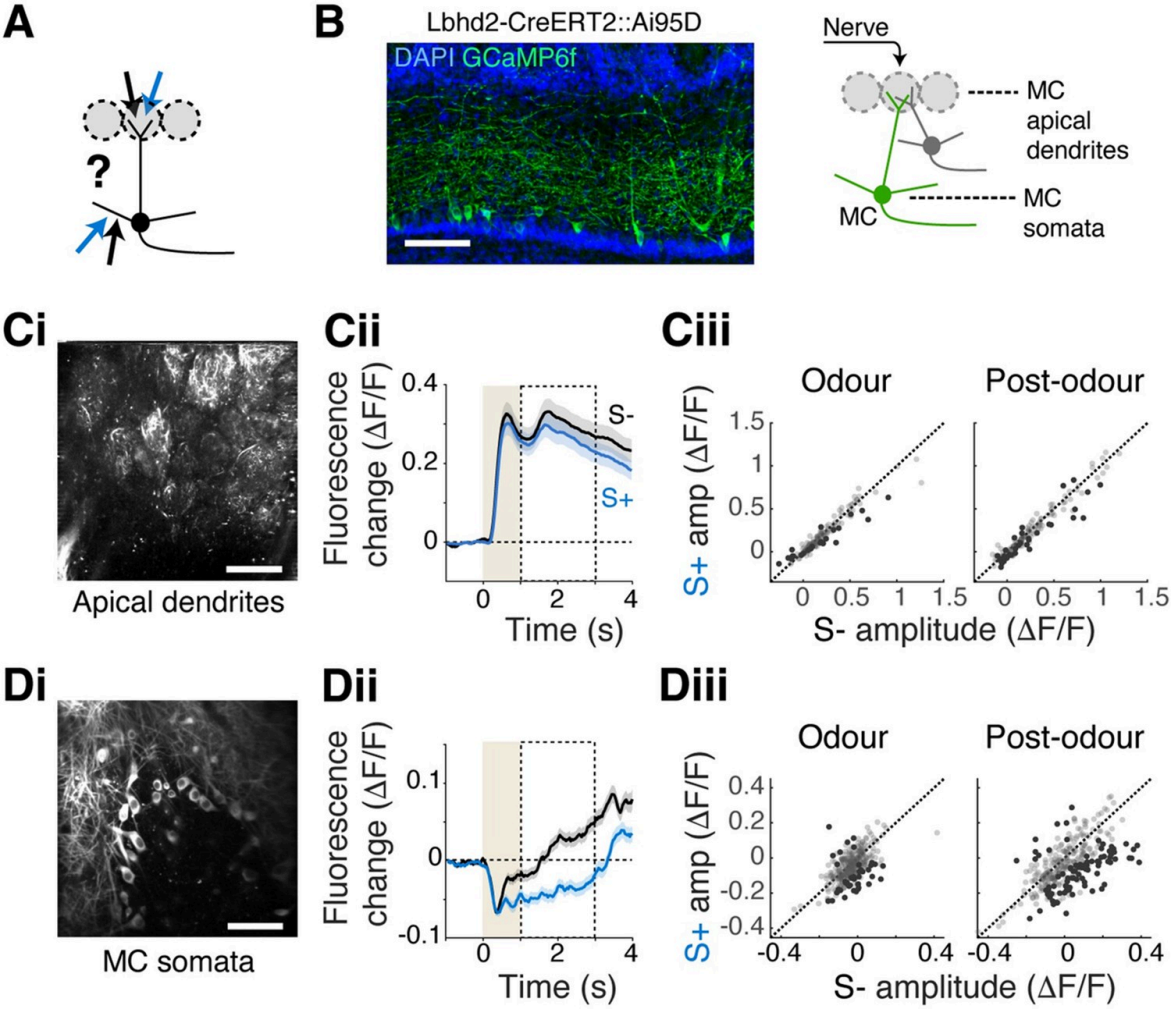
Reward-related inhibition originates perisomatically. (**A**) Schematic showing possible subcellular compartments where divergent signals may arrive, namely, apical dendrites in the glomerulus (upper arrows), and deeper, lateral dendrites (lower arrows). (**B**) Left, confocal image showing GCaMP6f preferentially in mitral cells (MCs) in Lbhd2-CreERT2::Ai95D mice. Right, illustration of imaging planes to obtain signals from the MC apical dendrites and somata. Scale bar = 100 μm. (**C**) Analysis of GCaMP6f signals from MC apical dendrites. (**Ci**) Example field of view from the glomerular layer. (**Cii**) Average fluorescence change from all ROIs (glomeruli) in response to S+ and S- odours. (**Ciii**) Comparison of average GCaMP6f fluorescence change for individual ROIs evoked by S+ vs. S- odours for the periods indicated. Darker points represent significantly divergent responses. (**Di–iii**) Same as **Ci–iii**, but for MC somata. Scale bar = 50 μm. *N* = 140 ROIs, 4 mice for apical dendrites and 321 ROIs, 7 mice for somata. Source data can be found in Fig 6 data, Dryad.

The results so far indicate that the widespread inhibitory responses associated with the rewarded odours come from sources extrinsic to the olfactory bulb. One of the major targets of such long-range projections within the olfactory bulb is the granule cells. These cells contact mitral cells on their lateral dendrites at a deeper portion of the external plexiform layer, although other inhibitory neurons contact mitral cells in the deeper subcellular compartments as well [47,48]. If the granule cells convey the contextual signals to mitral cells, the divergent responses may be observable perisomatically, but not in the superficial compartment (**Fig 6A**). To test this, we compared the GCaMP6f signals from the apical dendrites of mitral cells in the glomeruli versus signals from the somata, which reflect signals derived from all subcellular compartments. Since tufted cells and mitral cells both send their apical dendrites to the glomeruli, to study signals from mitral cells in isolation, we used Lbhd2-CreERT2::Ai95D mice, where GCaMP6f is expressed predominantly in mitral cells [33] (**Fig 6A and 6B**).

Imaging from the superficial plane, the apical dendrites showed no significant differences between responses to S+ and S- odours (mean ΔF/F during odour = 0.307 ± 0.439 and 0.328 ± 0.466 for S+ and S-, respectively; *p* = 0.687; post-odour = 0.338 ± 0.512 and 0.382 ± 0.549 for S+ and S-, respectively; *p* = 0.423, Wilcoxon rank-sum test, **Fig 6C**). As before, signals from the mitral cell somata imaged in the Lbhd2-CreERT2::Ai95D mice were characterised by the widespread inhibitory component (mean ΔF/F during odour = −0.058 ± 0.077 and −0.034 ± 0.061 for S+ and S-, respectively; *p* = 1.86 × 10^−4^; post-odour = −0.024 ± 0.130 and 0.038 ± 0.131 for S+ and S-, respectively; *p* = 1.08 × 10^−5^, Wilcoxon rank-sum test; **Fig 6D**). Divergent responses were also observed in the lateral dendrites in the vicinity of mitral cell somata (**S7 Fig**). Together, these data suggest that the reward-related inhibition in mitral cells originates perisomatically.

**Fig 7:**
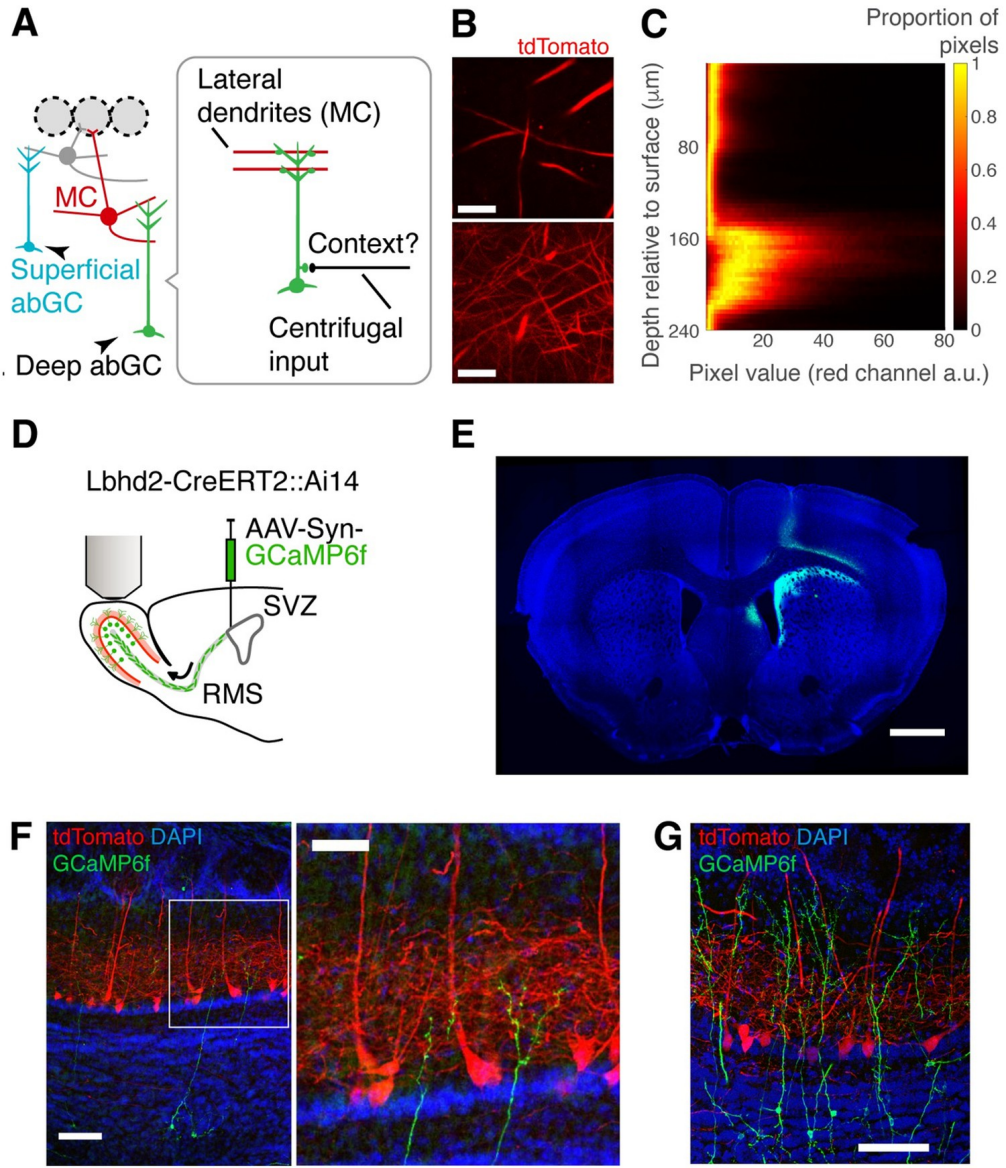
An experimental approach to image from superficial and deep adult-born granule cells. (**A**) Schematic of local circuitry and hypothesis; mitral cells (MCs) synapse with granule cells (GCs) whose dendrites ramify in the lower portion of the external plexiform layer. The deep-ramifying granule cells may receive the contextual signal that leads to the divergent responses in MC somata. (**B**) Lower and upper portions of the external plexiform layer can be distinguished by the density of MC dendrites. tdTomato is preferentially expressed in MCs in Lbhd2-CreERT2::Ai14 mice. Scale bars = 30 μm for top and bottom images. (**C**) Depth-dependent pixel intensity histogram from an example z-stack obtained with a two-photon microscope in a Lbhd2-CreERT2::Ai14 mouse. (**D**) Adult-born granule cells (abGCs) are made to express GCaMP6f by injecting AAVs in the subventricular zone (SVZ). Adult-born granule cells are imaged 4 weeks after injection. RMS = rostral migratory stream. (**E**) An example confocal image showing the site of AAV injection targeted to the lateral wall of the subventricular zone. Scale bar = 1 mm. (**F**) Example confocal images showing amplified GCaMP6f signal from deep abGC (green), shown with tdTomato signals (red) from MCs and DAPI signals (blue). Scale bars = 100 μm and 50 μm for left and right images, respectively. (**G**) Another example confocal image from a separate animal showing a mixture of adult-born GCs with deep and superficial dendrites. Scale bar = 100 μm.

If the inhibition in response to the rewarded cue in mitral cells is mediated via granule cells, we should observe a greater GCaMP6f signal change to S+ odours specifically in the granule cells that target mitral cells (**Fig 7A**). The granule cells whose dendrites ramify in the deeper portion of the external plexiform layer are thought to synapse with mitral cells [25,49,50], where mitral cell lateral dendrites are found. These mitral cell-targeting granule cells are, however, present intermixed with tufted cell-targeting granule cells.

To distinguish the putative mitral cell-targeting granule cells from those that target tufted cells, the depth of the external plexiform layer needs to be distinguished accurately *in vivo*. Towards this end, we crossed Lbhd2-CreERT2 mice with Ai14 mice to express tdTomato preferentially in mitral cells. We reasoned that despite the tissue curvature or non-uniform thickness of the external plexiform layer, this method would allow us to accurately separate the deeper portion from the superficial portion based on the density and distribution of the tdTomato expression. Indeed, the deep portion of the external plexiform layer showed higher density of thin red fluorescent processes (**Fig 7B and 7C**), while at more superficial depths, we observed occasional fluorescence from thick processes, likely corresponding to the primary dendrites of mitral cells.

To study if the divergent odour responses in mitral cells can be explained by the evoked activity of putative mitral cell-targeting granule cells, we turned to adult-born granule cells that develop their dendrites in the deep external plexiform layer (**Fig 7D and 7E**). Adult-born granule cells are thought to be critical for refining odour responses in mitral and tufted cells when mice need to discriminate between similar odours [51–55]. Further, since the mature adult-born granule cells form dendro-dendritic synapses with mitral cell lateral dendrites, where GABA release can occur locally [56], we sought to image directly from dendritic gemmules. Due to their small size, we were cautious to exclude images from sessions that showed motion artefact, which was determined by correlating the structural fluorescence pattern to the baseline period and discarded those that showed low correlation (**S8 Fig**). As a result, 70% (1,343/1,917 trials) of the acquired data was discarded.

**Fig 8:**
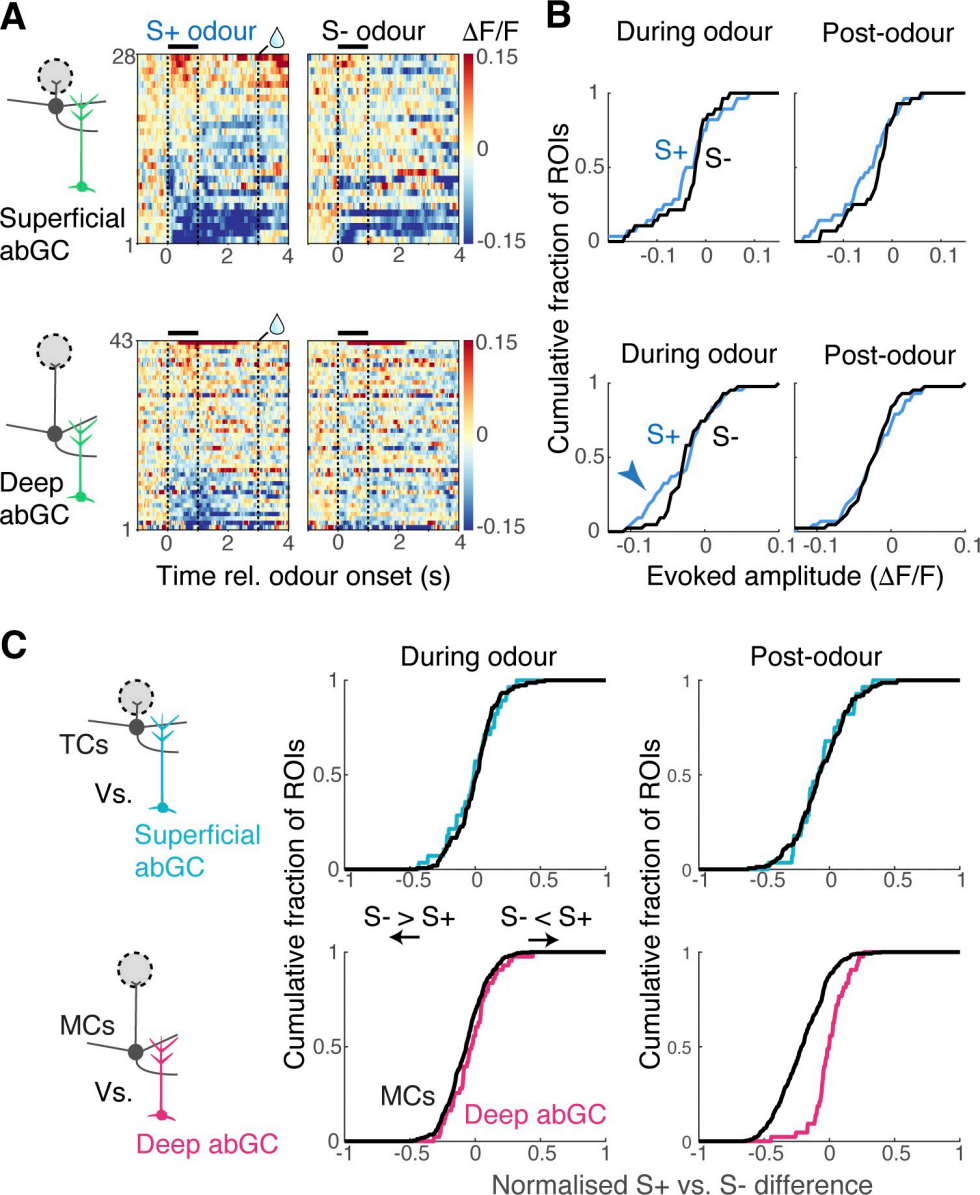
Imaging from deep adult-born granule cells suggests an extrinsic drive for mitral-targeting granule cells. (**A**) Colormap representation of normalised fluorescence change around the time of S+ and S- odour presentations (left and right, respectively) for the superficial gemmules (top) and deep gemmules (bottom). *N* = 28 and 43 ROIs for superficial and deep gemmules, respectively. (**B**) Comparison of S+ vs. S- response difference distributions for superficial abGC gemmules (top) and deep abGC gemmules (bottom). (**C**) Left, comparison of S+ vs. S- response difference distributions for tufted cell (TC) somata (black trace) and superficial gemmules (light blue); right, comparison of S+ vs. S- response difference distributions for MC somata (black trace) and deep gemmules (magenta). Source data can be found in Fig 8 data, Dryad.

We first characterised how the deep versus superficial dendritic gemmules respond to the rewarded versus unrewarded odours as mice performed difficult discrimination (**Fig 8A and 8B**). Inhibitory responses were generally more prevalent than excitatory responses in both cases, for both odours. In the deep gemmules, in the early phase, we observed slightly more inhibitory responses for the rewarded odour (**Fig 8A and 8B**). However, in the late phase, the S+ and S- response distributions almost completely overlapped. We wished to understand how well these granule cell dendritic responses could be explained by the local presynaptic counterparts, that is, against the distribution of evoked responses in mitral cells and tufted cells. The S+ versus S- tuning showed a close overlap between tufted cells and superficial gemmules of adult-born granule cells (**Fig 8C**). On the other hand, S+ versus S- tuning distribution of deep gemmules during the late phase could not be explained by the mitral cell tuning distribution (**Fig 8C**). There was a tendency for these gemmules to respond more positively to the rewarded odours than would be predicted from mitral cell activity alone. In other words, our data suggests that mitral cell-targeting granule cells may receive an additional excitatory drive associated with the rewarded odour.

## Discussion

In this study, we observed a cell type-specific reward-associated inhibition in the primary olfactory area of the mouse. This inhibition is cell type specific and subcellular specific: first, it manifests in mitral cells but not in tufted cells, and second, it appears in the somata but not in their apical dendritic tuft in the input layer. This subcellular specificity suggests that the generation of this phenomenon involves circuit components at a deeper layer of the olfactory bulb. Further, the results of pseudo-conditioning and pharmacological manipulations suggest that the mitral cell-specific, reward-related inhibition arises from an acquired congruence of sensory and contextual signals. Our study reinforces the idea that value-related modulation of olfactory signals is a key aspect of primary olfactory processing, and narrows down potential underlying mechanisms by identifying deeper circuit components that perisomatically interact with mitral cells.

Note that we refer to a reduction in the GCaMP6f fluorescence relative to the baseline as “inhibition.” While such a fluorescence reduction is known to correlate with hyperpolarisation in the membrane potential [57] or a reduction in extracellularly recorded spike numbers [58], electrophysiology would be needed in a future experiment to confirm if indeed a reduction in the mitral cells’ output is widespread in the late-phase responses to reward-predictive odours.

Questions remain regarding the origin of the reward-related signals to the olfactory bulb. Many brain regions send long-range projections to the olfactory bulb and are, therefore, candidate drivers of the reward-related inhibition we observed. One important source of reward-related signals to the olfactory bulb is the direct or indirect feedback projections from olfactory cortices. Value-like modulation of olfactory responses occurs in many parts of the brain: It has been observed in the prefrontal cortex [4,59], orbitofrontal cortex [4,59], hippocampus [60], olfactory tubercle [61–64], piriform cortex [4,46], and anterior olfactory nucleus [4], although there may be regional differences, for example, in the long-term stability of expression [59]. Of particular interest is the piriform cortex, which serves as a gateway for processed signals for modulation of the mitral cells [17]. Indeed, reward-related activity has been observed in the anterior piriform cortex especially in the late phase [46], though not in the posterior piriform cortex [62,64]. While only a subset of pyramidal neurons from the piriform cortex project to the olfactory bulb [65], a recent imaging study from olfactory bulb-projecting fibres showed value-like activity when the task depended on olfactory cues [66]. It should be noted that the muscimol infusion may have disrupted the functions of neighbouring or downstream areas, which include the olfactory tubercle and the striatum. That is, the muscimol-induced reduction in the reward-related inhibition could have been indirect. Future experiments will be needed to distinguish the role played by the piriform cortex more carefully, for example, by silencing the feedback fibres to the olfactory bulb locally [66]. It is unclear why we did not observe the widespread reward-related modulation in tufted cells, even though some value-like activity is present in the anterior olfactory nucleus [4], a region known to have modulatory influence over tufted cells [17]. Since the anterior olfactory nucleus has multiple compartments [67], each with distinct long-range connectivity [68], it will be crucial for future studies to resolve how these subregions contribute to associating values with olfactory stimuli.

Another potential source of reward-related signals is classical neuromodulatory regions. Of particular interest are those that innervate the deeper layers of the olfactory bulb, including cholinergic neurons of the nucleus of the horizontal limb of the diagonal band [19,69], noradrenergic neurons of the locus coeruleus [70,71], and serotonergic neurons of the dorsal raphe nucleus [72,73]. These regions contain neurons that show reward-related signals, although they differ in the details. Cholinergic neurons of the basal forebrain are locked to the time of anticipatory behaviour in reward-driven sensory tasks [74,75]. This timing is relatively late compared to our phenomenon. In addition, optogenetic activation of cholinergic fibres in the olfactory bulb enhances, rather than inhibits, odour-evoked responses in mitral and tufted cells [19]. Normal adrenergic transmission within the olfactory bulb is required for learning to discriminate similar odours [76,77]. Neurons of the locus coeruleus, too, show instances of reward-locked activations [78]. As with acetylcholine, however, the timing is locked more to the anticipatory actions and further, modulation may enhance, rather than inhibit, olfactory responses in the olfactory bulb during delay conditioning 22. Therefore, cholinergic and adrenergic inputs may not relate directly to the phenomenon described in this study. On the other hand, among serotonergic neurons of the dorsal raphe nucleus, some lock to rewarded cues [79–81], making the serotonergic input a promising candidate for the reward-related inhibition in mitral cells. Precisely what the serotonergic signal represents remains an active area of research, with possibilities including reward [80], uncertainty [81], or a motor-sensory variable [82], though this may reflect regional differences, too [81,83]. Since serotonergic neurons also target the striatum to affect the dopamine release in the region [84], future experiments must resolve if they pose direct reward-related effects on the olfactory bulb or via other brain areas.

The value-like activity in the higher olfactory areas mentioned above appears as excitatory responses to the rewarded odour. In contrast, mitral cells of the olfactory bulb showed enhanced inhibitory responses. This is reminiscent of a human study, where perceived unpleasantness (negative valence) positively modulated the late component of odour-evoked responses in the olfactory bulb [85]. The sign reversal in the olfactory bulb suggests an involvement of inhibitory interneurons like the granule cells. Due to the perisomatic nature of the reward-related inhibition, the granule cells are a prime candidate. They are the major recipients of long-range projections in the olfactory bulb and may target mitral cells versus tufted cells separately by ramifying dendrites at the deep versus superficial levels of the external plexiform layer [25,49,50]. Despite their numerical dominance [86], how granule cells contribute to signal transformation in the olfactory bulb remains enigmatic. They are thought to contribute to the temporal precision of the olfactory bulb output [29,87,88], which is perceptible to the animals [89]. Granule cells seem to contribute only subtly to mitral and tufted cells’ spontaneous and evoked firing rates under anaesthesia or when mice are not engaged in behavioural tasks [29]. However, they can potently silence mitral and tufted cells or accelerate perceptual learning when activated in bulk [29,90]. As a result, it has been hypothesised that an excitatory drive from long-range projections onto granule cells is critical for potent physiological effects [91]. Because feedback projections synapse more proximally to the soma, these have more significant electrical impacts at the soma than inputs from the mitral and tufted lateral dendrites arriving more distally. This drive may be critical to a global activation of these neurons [91], serving as a potential associative mechanism [56]. Our depth-specific imaging approach may open new ways to investigate the physiology of these enigmatic interneurons.

Feedback signals from higher sensory regions may allow refined or processed information to be integrated into more peripheral processing, as in the olfactory bulb. Such a system may be used to dynamically tune the nature of early sensory processing to the behavioural demands at hand. But given the late timing we observed, where the reward-related inhibition is most visible during the post-odour period, which is after decisions have already been made, feedback signals may be used to fine-tune the process of maturation of adult-born granule cells and their integration into the existing circuitry within the olfactory bulb in a behaviourally relevant manner [51–53]. We speculate that the enhanced late inhibitory component for a more difficult olfactory discrimination may correspond to a mechanism to decorrelate similar olfactory response patterns [90,92,93]. It is possible that this occurs in a reward-dependent manner. It will be an intriguing future investigation to test if disruption of feedback signals prevents the proper establishment of acquired connectivity and, as a result, the expression of task-dependent activity patterns. In summary, our work brings us closer to a mechanistic understanding of context-dependent modulation involving a congruent interaction between local and long-range inputs.

## Materials and methods

### Animals

All animal experiments had been approved by the Okinawa Institute of Science and Technology Graduate University Graduate Animal Care and Use Committee (Protocol 2020–310). Tbx21-Cre [36], B6J.Cg-Gt(ROSA)26Sortm95.1(CAG- GCaMP6f)Hze/MwarJ, also known as Ai95D [94], and Ai14 [95] mice were originally obtained from the Jackson Laboratory (stock numbers: 024507, 028865, and 007914, respectfully). Lbhd2-CreERT2 mice were generated previously and are also available through Jackson Laboratory (stock number 036054; [33]). Tbx21-Cre::Ai95D and Lbhd2-CreERT2::Ai95D mice were generated by crossing parents homozygous for each transgene. Lbhd2-CreERT2::Ai14 mice were generated by crossing Lbhd2-CreERT2 mice with Ai14 mice.

### Tamoxifen administration

To induce Cre-recombinase activity in Lbhd2-CreERT2::Ai95D and Lbhd2-CreERT2::Ai14 mice, tamoxifen injections (3 × 80 mg/kg at p21, s.c.), or tamoxifen diet (553.8 ± 362.6 mg/kg at p21) was used. For injections, each of 3 consecutive days, tamoxifen solution (8 mg/ml; Sigma-Aldrich T5648) was used. Tamoxifen powder was first dissolved in absolute ethanol. This was then added to corn oil, resulting in a 5% ethanol and 95% corn oil mixture, and heated at 65°C on a shaker for 30 min. After it cooled down to room temperature, the solution was injected intraperitoneally (approximately 100 μl). Mice that were treated with the tamoxifen diet (2 mg/kg) were exposed to this for 2 to 4 days, based on their initial weight, after which they were switched back to normal food (threshold to switch to normal food: 80% initial body weight). Tamoxifen intake was calculated based on the amount of diet food provided and amount of diet food left after switching back to normal food.

### Surgery

All recovery surgeries were conducted in an aseptic manner. For the cranial window and headplate implantations, 9- to 11-week-old male mice were deeply anaesthetised with isoflurane (3% to 5% for induction, 1% to 2% for maintenance; IsoFlo, Zoetis Japan). A craniotomy of approximately 1.5 × 1 mm was performed over the left olfactory bulb, and a custom cut glass window (thickness No. 1; Matsunami, Japan) was implanted. Once the window was sealed with cyanoacrylate (Histoacryl, B. Braun, Germany), a custom-made metal headplate (26 × 12 mm) was implanted posterior to the cranial window. Dental acrylic (Kulzer, Hanau, Germany) was then added to cover the exposed skull and to secure both the headplate and cranial window.

For experiments involving pharmacological infusion, an additional cannula (10 mm length; C315GS- 4/SPC, Plastics One) was inserted to target the left anterior piriform cortex (coordinate: AP -2.2 mm and ML -2.4 mm from bregma; DV -6.1 mm at 45-degree angle from brain surface), similar to [18].

All mice were administered carprofen (5 mg/kg, i.p.) post-operatively for 3 consecutive days. All mice were recovered for at least 2 weeks before the start of behavioural experiments.

### Virus injection

To express GCaMP6f in adult-born granule cells, during cranial window and headplate implantations, Lbhd2-CreERT2::Ai14 mice were injected with AAV1-syn-GCaMP6f-WPRE-SV40 (Addgene 100837-AAV1; titre was 1.84 × 10^13^ GC/ml at the time of synthesis) in the left SVZ (200 μl; coordinate: AP 1.0 mm and ML -1.0 mm from bregma; DV -2.2 mm vertically) using Nanoject III (Drummond, 3-000-207).

### Habituation

Male mice were habituated to head fixation on a custom-made running wheel. Thereafter, water access was restricted by removing water bottles from their home cages. The mice were habituated to receive water from the port at the experimental setup on the following 2 to 3 days until they learned to drink at least 1 ml at the setup. The body weight was recorded daily to ensure that it stayed above 80% of the original weight. Lick responses were measured using an IR beam sensor (PM- F25, Panasonic, Osaka, Japan).

During calcium imaging sessions, respiration of the mice was recorded using a flow sensor (AWM3100V, Honeywell, North Carolina, United States of America) placed close to the right nostril.

### Discrimination training

After habituation, the mice were trained to associate 1 odour stimulus with a water reward (S+ odour) and another odour stimulus with no reward (S- odour). Both S+ and S- odours were a binary mix of ethyl butyrate (Sigma-Aldrich; W242705) and methyl butyrate (Tokyo Chemical Industry; B0763), but mixed with different ratios based on the photoionization detector readings. Odour presentation was targeted to the left nostril since the right nostril was used to record the nasal airflow with a flow sensor. For the initial, easy, discrimination training, an 80/20 versus 20/80 ratio was used. When mice reached 80% behavioural accuracy, difficult discrimination training started, using 60/40 versus 40/60 odour mixtures. The correct response to S+ odours was to lick within a 3-s window after stimulus onset, while the correct response to S- odours was to refrain from licking. The water reward consisted of multiple drops, with a total of approximately 18 μl per trial. Olfactory stimuli were presented using a custom flow-dilution olfactometer [96]. On each trial, odour was presented for 1 s and delay to the reward was 3 s from the odour onset. Inter-trial interval was approximately 20 s.

### Long odour discrimination

The mice proficient at the difficult discrimination task were trained to discriminate between the same odour mixtures but with 4 s of odour duration. The response window and reward timing on S+ trials was the same between the 2 paradigms.

### Disengagement paradigm

In this paradigm, the same odour mixtures as the difficult discrimination were used, but the water reward was delivered every trial, approximately 15 s before the odour onset. The time window used for measuring the anticipatory licks was identical to that of the difficult discrimination paradigm. The first session was considered a transition session and excluded from imaging analysis.

### Random association paradigm

In this paradigm, the water reward was presented in 50% of the trials, regardless of the odour identity. This decoupled the odour identity and reward, but kept mice engaged, as indicated by the anticipatory licks. The first session was considered a transition session and excluded from imaging analysis.

### Pharmacological inactivation of anterior piriform cortex

For pharmacological inactivation of the anterior piriform cortex, muscimol (M1523, Sigma-Aldrich, Missouri, USA) was infused (2 mM in Ringer; 500 nL at 100 nL/min) through the previously implanted cannula, using a Hamilton syringe (1 μl Model 7001KH PST-3 80100, Hamilton Company, Nevada, USA), approximately 10 min before the start of the imaging sessions. Two days prior to the first muscimol infusion, Ringer solution (500 nL at 100 nl/min) was infused.

### Histology

After the conclusion of the behavioural experiments, the mice were perfused transcardially with phosphate buffer (in mM): NaH2 PO4 (225.7), Na2HPO4 (774.0) with pH adjusted to 7.4, followed by PFA solution (4% dissolved in phosphate buffer). For mice that were implanted with a cannula, 500 nL DiI (Invitrogen, V22885) was injected prior to the perfusion to mark the cannula tip location. Coronal sections of 100 μm thickness were cut on a vibratome (5100 mz-Plus, Campden Instruments, Leicestershire, United Kingdom) and counterstained using DAPI (D9542, Sigma-Aldrich). Images were acquired using a Leica SP8 confocal microscope using a ×10 (NA 0.40 Plan-Apochromat, 506407, Leica) objective.

### Immunohistochemistry

Free floating olfactory bulb sections from above were first blocked in blocking solution (0.025 M Tris-HCl, 0.5 M NaCl, 0.2% triton X-100, 7.5% normal goat serum, 2.5% BSA, pH = 7.5) for 60 min at room temperature. Slices were subsequently stained with chicken anti-GFP (Abcam, ab13970; 1:500 in blocking solution) at 4°C overnight. Slices were washed 3 times in TBS (0.025 M Tris-HCl, 0.5 M NaCl, pH = 7.5) and incubated in goat anti-chicken Alexa-488 (Abcam, ab150169; 1:1,000 in TBS supplemented with 0.2% triton X-100) for 2 h at room temperature. All slices were counter stained with DAPI (D9542, Sigma-Aldrich). Images were acquired using a Leica SP8 confocal microscope using a 10× (NA 0.40 Plan-Apochromat) or a 40× (NA 1.10 Plan-Apochromat, 506357, Leica) objective.

### In vivo calcium imaging

All the calcium data presented in this manuscript were obtained from awake mice. Two-photon fluorescence of GCaMP6f and tdTomato were measured simultaneously with a custom-made microscope (INSS, UK) fitted with a 25× objective (Nikon N25X-APO-MP1300, 1.1 N.A.) or a 16× objective (Nikon N16XLWD-PF, 0.8 NA), and high-power laser (980 nm; Insight DeepSee, MaiTai HP, Spectra-Physics, USA) at depths 50 to 400 μm below the surface of the olfactory bulb. Images from a single plane were obtained at approximately 30 Hz with a resonant scanner. In each trial, 400 image frames were acquired, with 100 frames before odour stimulus to obtain a baseline. Each day, the stage coordinates were chosen relative to a reference location, which was determined by the surface blood vessel pattern. Fields of view were 512 μm × 512 μm for apical dendrites, 256 μm × 256 μm for tufted and mitral cell somata, and 128 μm × 128 μm and for adult-born granule cell gemmules. Calcium data during difficult discrimination and disengaged experiments were obtained from Tbx21-Cre::Ai95D mice (Figs 1 and 4). Six male mice were used for somata imaging. All 6 were used to image mitral cell somata, while a subset (3 mice) were used to image tufted cell somata. To obtain calcium data from different subcellular compartments of mitral cells, we used Lbhd2-CreERT2::Ai95D mice (**Fig 6**). For long odour discrimination, random association, and muscimol infusion experiments, calcium data was obtained from both Tbx21-Cre::Ai95D and Lbhd2-CreERT2::Ai95D mice (**Figs 3**–**5**). Finally, Lbhd2-CreERT2::Ai14 mice were used in to record red (tdTomato, mitral cells) and green (calcium indicator, gemmules) fluorescent signals during adult-born granule cell imaging experiments (**Figs 7** and **8**). The “superficial” and “deep” levels were approximately 40 to 50 μm above and below from the transition in the red fluorescence density, respectively.

### Data analysis

All data was analysed offline using custom MATLAB (MathWorks, USA) routines. To calculate the behavioural accuracy, the number of licks during a 3-s window from final valve opening until reward presentation was counted for each trial (anticipatory licks). Correct response to the rewarded odour was a minimum of 2 anticipatory licks, and correct response to the unrewarded odour was less than 2 anticipatory licks. Behavioural accuracy was calculated as the percentage of correct trials from the total number of trials.

To calculate the sniffing frequency and speed of inhalation, the sniffing signal was first filtered (1 Hz high-pass and 30 Hz low-pass) and normalised (z-score). Inhalation peaks were detected using the *findpeaks* MATLAB function. Sniff onsets were determined by searching back in time from each detected inhalation peak to the point where the signal crossed a threshold value. The detected onsets and peaks were then used to calculate the frequency (as 1/inter-onset time) and speed of inhalation (as onset-to-peak time).

### Image analysis

For each field of view, the imaging data was manually curated based on motion artifacts and drift over time. Data with motion artifacts and/or drift were motion corrected using the NoRMCorre toolbox [97] and, when unsuccessfully corrected, excluded from analysis. ROIs were manually drawn using ImageJ (NIH, Bethesda, USA) based on the average field of view from each imaging session and exported for usage in MATLAB. Average pixel value from each ROI was offset with a value from the darkest region in the frame (e.g., a blood vessel). To account for bleaching over the course of the imaging session, the mean pixel values for all trials were concatenated and detrended using the MATLAB function *detrend*, then reshaped back into an array (individual trials × frames) before relative fluorescence change was obtained. For each ROI, the change in fluorescence (ΔF/F) was calculated by subtracting the mean pixel value from the baseline period (1 s before odour stimulus onset) and dividing by the baseline value. Odour-evoked responses were calculated as the mean fluorescence change during the odour stimulus presentation, and post odour-evoked responses as the mean fluorescence change between odour stimulus offset and reward presentation. For the “long odour” and “random association” experiments, the time windows to calculate the evoked responses were based on the difficult odour discrimination experiments. All visible ROIs from each field of view are included in the plots unless otherwise stated.

### Lick-aligned average

Rewarded trials were analysed for each imaging session. Onsets of anticipatory licks were defined as the average time of the first 2 licks observed after the start of odour presentation. Rewarded trials were grouped into early versus late lick trials if the anticipatory lick onsets occurred before or after the median onset time, respectively. Within each group, calcium transients were aligned to the anticipatory lick onset time for each rewarded trial and averaged. Note that the reaction time considers only the timing of lick onsets and is distinct from the discrimination time which considers the time at which S+ versus S- responses diverge.

### Quality assessment abGC imaging

Small abGC gemmules make them susceptible to motion artifacts in behaving animals. To objectively assess the quality of the imaged trial, the tdTomato signals from the MC dendrites in Lbhd2-CreERT2::Ai14 mice were analysed. Rolling averages of 5 frames (step size: 1 frame) were made and compared against the average of 50 frames obtained during the baseline period to compute the correlation coefficient. If the mean correlation value for the period analysed (between the odour offset and onset of water reward) was below 95% of the mean value during the baseline period, the trial was rejected. This quality check was conducted separately for the odour and post-odour phase, and only those trials that met the quality in both phases are included in the analysis. Further, trials where the baseline correlations deviated significantly were considered outliers and rejected. This was assessed using the Matlab function *isoutlier*. This quality control method resulted in 16 accepted fields of view from 5 mice for the odour period and 13 fields of view from 4 mice for the post-odour period. On average, a given accepted fields of view yielded 3.2 ± 1.4 ROIs (3.8 ± 1.5 ROIs for deep FOVs, and 2.9 ± 1.4 ROIs for superficial fields of view for the magnification used (128 μm × 128 μm frame size)).

### External plexiform layer depth determination based on red fluorescence

Depth within the external plexiform layer was estimated using the red fluorescence signal from mitral dendrites in Lbhd2-CreERT2::Ai14 mice, which is dense in the deeper portion. Z-stack ranging from the superficial layer to mitral cell layer spanned 250 μm (100 frames averaged every 4 μm) obtained from the same x-y location as the functional imaging was used. Since the fibre-like structures are the relevant signals, the averaged frame from each depth was passed through a filter available as a plug-in in ImageJ (“Tubeness” [98]), with the sigma parameter set to 2 μm.

### Normalised S+ versus S- difference

For each trial, the average value of relative fluorescence change was calculated for the odour period (first 1 s after the odour onset) and the post-odour period (1 to 3 s after the odour onset). The normalised difference between S+ and S- response amplitudes for the odour period, as well as the post-odour period, is calculated as follows:

$$\frac{\left(\frac{\sum_{i}^{n}x_{i}}{n}-\frac{\sum_{i}^{n}y_{i}}{n}\right)}{\left(max(x)+max(y)\right)},$$

where *i* denotes the trial index, *n* is the number of trials, x is the evoked fluorescence change in response to the rewarded odour, and y is the evoked fluorescence change in response to the unrewarded odour.

### Statistics

#### Significant responses

For each ROI, whether an evoked response is significantly different from the baseline was determined by comparing the mean evoked fluorescence change against the mean and standard deviation of the fluorescence fluctuations during the baseline period, which was the 1.5 s period before the onset of the odour. Significant responses were those that deviated from the baseline by more than 3 standard deviations.

#### Divergent responses

To determine if an ROI showed a divergent response, for each ROI odour-evoked and post-odour response amplitudes for S+ and S- trials were tested for statistical significance using the Wilcoxon rank sum test. Summary transients presented in figures show mean ± SEM, unless otherwise stated.

#### Discrimination time

The method for determining the discrimination time was modified from [99]. Cumulative histograms of the detected licks were calculated for all trials using 50 ms time bins. For each time bin, the histogram values for S+ trials and for S- trials were tested for statistical significance using a Wilcoxon rank sum test. The first time bin where histograms were significantly different (*p*-value less than 0.05) was taken as the discrimination time.

### Dryad DOI

https://doi.org/10.5061/dryad.r4xgxd2m9 [100].

## Supporting information

S1 Fig Time-course of decision-related behavioural output. (**A**) Example raster plots showing lick times relative to the onset of odour (t = 0) and reward delivery (t = 3 s) of a proficient mouse. Trials have been sorted into rewarded (S+) and unrewarded (S-) trials. Whether the mice made the correct or incorrect decision was determined based on the number of licks observed between the odour onset and reward onset. Green ticks on the right indicate correct trials, and red ticks indicate incorrect trials. (**B**) Calculation of discrimination time is the earliest time at which licking patterns for S+ and S- trials diverge significantly, at the 0.05 level, shown for the example session in **A**. (**C**) Discrimination time for all mice presented in Fig 1. *N* = 17 sessions, 6 mice.
(TIF)

S2 Fig Sniff patterns are not starkly different between rewarded vs. unrewarded trials. (**A**) Example sniff signal from a flow sensor placed next to a nostril on the contralateral side to odour presentation. Upward signal (signal above dotted horizontal line) corresponds to inhalation. Inhalation peaks are shown with circles (top) and inhalation onsets are annotated with short vertical ticks (bottom). (**B**) Instantaneous sniff frequency is defined as the reciprocal of the sniff interval, measured from one inhalation onset to the next inhalation onset. Speed of inhalation (time to peak) is defined as the time elapsed from the inhalation onset to the inhalation peak. (**C**) Time course of instantaneous sniff frequency change relative to the odour period (light brown background) and post-odour period (demarcated with dotted lines). Mean and SEM shown. (**D**) Cumulative histogram of instantaneous frequencies observed during post-odour period. Thin lines correspond to individual sessions, and thick lines correspond to average across imaging sessions. (**E**) Same as **D**, but for inhalation speed. *N* = 13 sessions, 7 mice.
(TIF)

S3 Fig Statistics of evoked responses in mitral and tufted cells in mice proficiently performing the difficult olfactory discrimination task. (**A**) Proportion of ROIs that respond with significant inhibition (leftward bar) and excitation (rightward bar) for mitral cells (grey) and tufted cells (black). (**B**) Proportion of ROIs that show significant divergence in response between S+ and S- odours. Odour period is during the 1 s odour presentation, while post-odour period corresponds to 1–3 s after the odour onset (0–2 s after the odour offset), before the reward delivery.
(TIF)

S4 Fig Evoked responses tend to be more inhibited when animals anticipate reward. (**A**) Cumulative histograms of fluorescence changes during the odour (left) and post-odour (right), averaged over “Hit” trials (mice generate anticipatory licks in response to the S+ odour; green) vs. “Miss” trials (mice failed to generate anticipatory licks after the S+ odour presentation; red). Data is from mitral cell somata of Tbx21-Cre::Ai32 mice performing the difficult discrimination. Bottom row: same plots but with the x axis ranges indicated above. (**B**) The observed difference in the median evoked amplitude of the “Miss” distribution was subtracted from the median evoked amplitude from the “Hit” distribution in **A** (“Hit-miss difference”) and was compared against a shuffled distribution, where trial indices were randomly permutated. The random permutation was repeated 10,000 times. (**C**, **D**) Same as **A**, **B**, but for the S- odour, and correct and incorrect outcomes correspond to “Correct rejection” and “False alarm,” respectively.
(TIF)

S5 Fig Reward-related, post-odour divergence in the mitral cell somatic responses is reduced with long odour presentation. (**A**) Schematic showing the time course of short vs. long odour presentations. Short odour presentation involved 1-s presentation of odours, followed by a 2-s trace period before the reward delivery. With the long odour presentation, a 4-s odour presentation overlapped in time with the reward delivery, which occurred at 3 s. (**B**) Cumulative histograms of S+ vs. S- response amplitudes (normalised by the maximum magnitude for the entire dataset) for the short odour (black) and long odour (orange) experiments.
(TIF)

S6 Fig Reward-related divergence in the late phase is present but reduced with easy discrimination task. (**A**) Schematic of the easy discrimination task. (**B**) Colormap representation of S+ and S- responses imaged from mitral cell somata in Lbhd2-CreERT2::Ai95D mice performing the easy task. Top, ROI indices were sorted by the S+ response amplitudes; bottom, ROI indices were sorted by the S- response amplitudes. (**C**) Average fluorescence change of all ROIs (mitral cell somata) for the S+ (blue) and S- (black) odours. (**D**) Scatter plot comparing S- vs. S+ responses for odour (top) and post-odour (bottom) periods. Each point represents 1 ROI, and the data shown are from all sessions and mice. Dotted line represents unity (S- amplitude = S+ amplitude). Individual points correspond to ROIs. Black dots indicate S+ and S- responses that were significantly different.
(TIF)

S7 Fig Reward-relate inhibition is observed in the lateral dendrites as well. (**A**) An example field of view from an Lbhd2-CreERT2::Ai95D mouse at the mitral cell layer. ROIs were manually drawn around the lateral dendrites proximal to the somata as illustrated in the schematic (left). (**B**) Normalised fluorescence change (ΔF/F) for the ROIs indicated in **A** around the time of the rewarded odour (left) and unrewarded odour (right). (**C**) Colormap representation of normalised fluorescence change (ΔF/F) for all ROIs. (**D**) Scatter plots comparing the amplitude of fluorescence change evoked by S+ odour vs. S- odour for the odour period (above) and post-odour period (below). Dots correspond to ROIs from all animals shown. Black dots indicate significantly divergent responses.
(TIF)

S8 Fig A method for assessing the quality of adult-born granule cell imaging. (**A**) Example field of view from the red channel showing mitral cell dendrites. (**B**) Image quality was determined by the frame-by-frame similarity of red fluorescence patterns by calculating correlation in the tdTomato image between the baseline period and other time points within the trial. Those with a high correlation coefficient throughout the trial is deemed to have less drift, e.g., due to animal’s movements. (**C**) Time course of red fluorescence correlation values for the accepted dataset. (**D**) Same as **C** but for the rejected dataset. Of the 1,917 trials imaged in total 574 trials were accepted and 1,343 trials were rejected. This amounts to, on average, 27.7% acceptance rate for deep gemmules and 36.2% acceptance rate for superficial gemmules.
(TIF)
