# Supplementary figures and images for: Value-related learning in the olfactory bulb occurs through pathway-dependent perisomatic inhibition of mitral cells
Source: PLoS Biol. 2024 Mar 1;22(3):e3002536. doi: 10.1371/journal.pbio.3002536 (PMC10936853; doi:10.1371/journal.pbio.3002536)

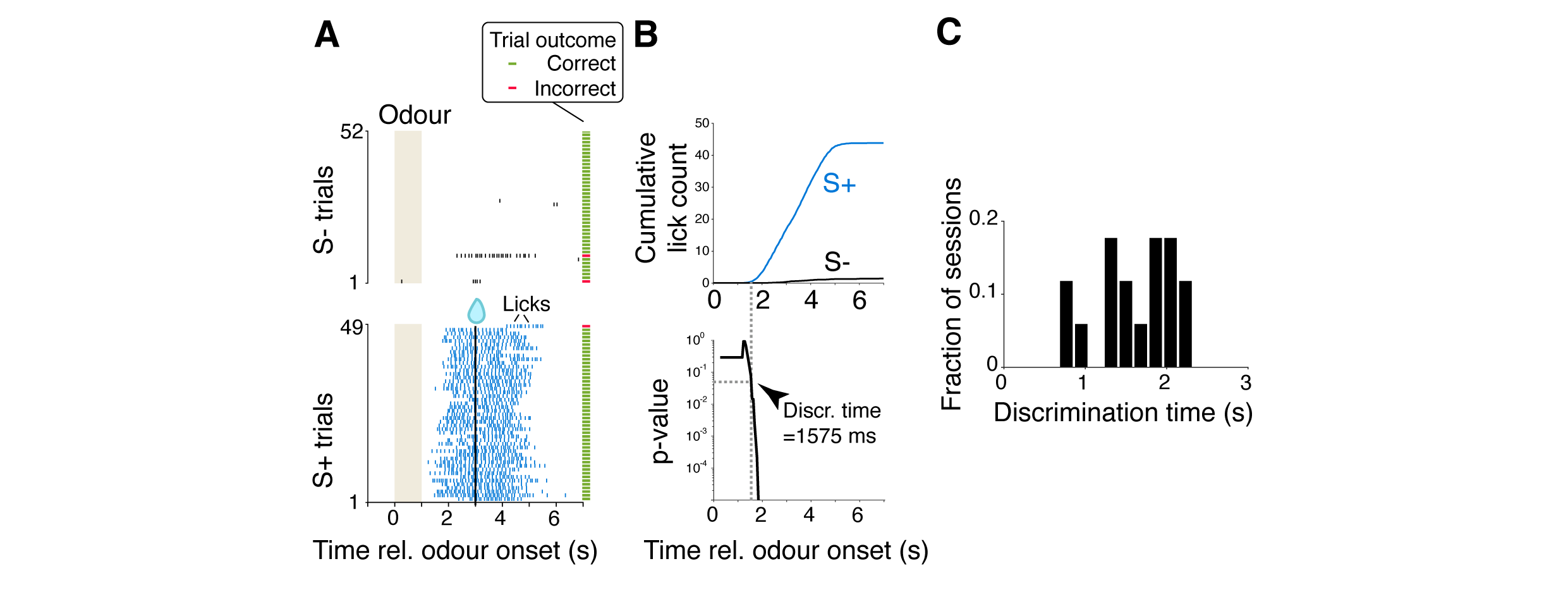

Supplement: S1 Fig — (A) Example raster plots showing lick times relative to the onset of odour (t = 0) and reward delivery (t = 3 s) of a proficient mouse. Trials have been sorted into rewarded (S+) and unrewarded (S-) trials. Whether the mice made the correct or incorrect decision was determined based on the number of licks observed between the odour onset and reward onset. Green ticks on the right indicate correct trials, and red ticks indicate incorrect trials. (B) Calculation of discrimination time is the earliest time at which licking patterns for S+ and S- trials diverge significantly, at the 0.05 level, shown for the example session in A. (C) Discrimination time for all mice presented in Fig 1. N = 17 sessions, 6 mice. (TIF) [file pbio.3002536.s001.tif]

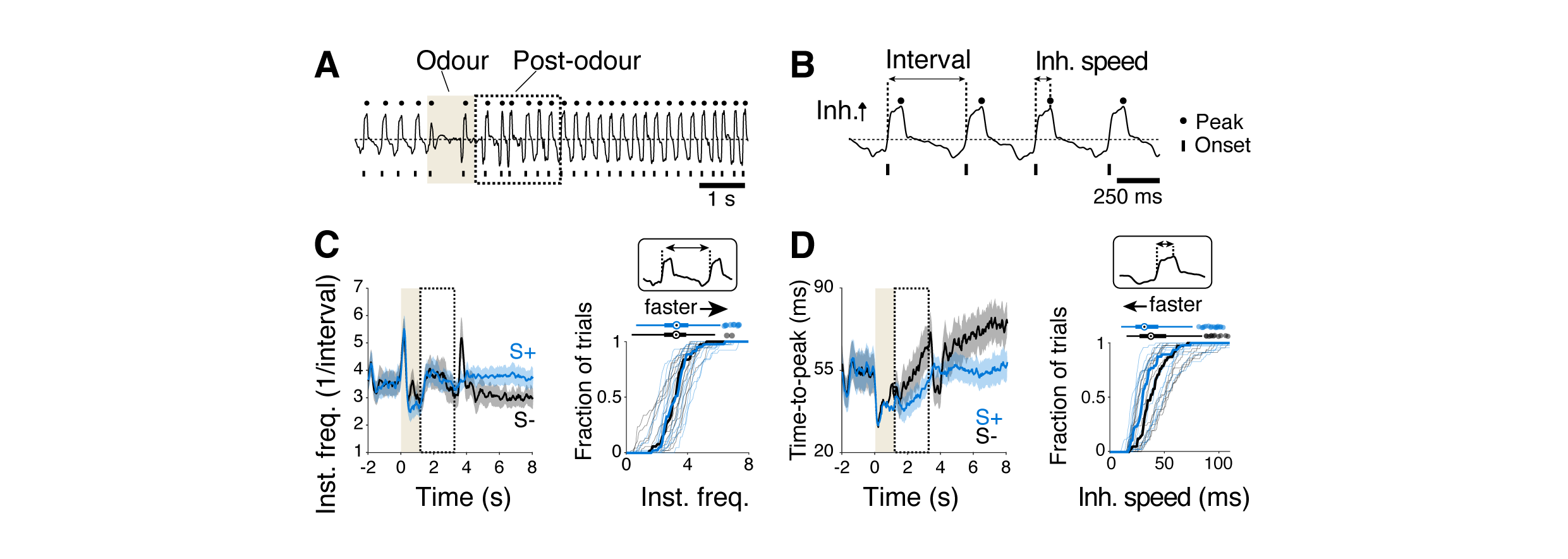

Supplement: S2 Fig — (A) Example sniff signal from a flow sensor placed next to a nostril on the contralateral side to odour presentation. Upward signal (signal above dotted horizontal line) corresponds to inhalation. Inhalation peaks are shown with circles (top) and inhalation onsets are annotated with short vertical ticks (bottom). (B) Instantaneous sniff frequency is defined as the reciprocal of the sniff interval, measured from one inhalation onset to the next inhalation onset. Speed of inhalation (time to peak) is defined as the time elapsed from the inhalation onset to the inhalation peak. (C) Time course of instantaneous sniff frequency change relative to the odour period (light brown background) and post-odour period (demarcated with dotted lines). Mean and SEM shown. (D) Cumulative histogram of instantaneous frequencies observed during post-odour period. Thin lines correspond to individual sessions, and thick lines correspond to average across imaging sessions. (E) Same as D, but for inhalation speed. N = 13 sessions, 7 mice. (TIF) [file pbio.3002536.s002.tif]

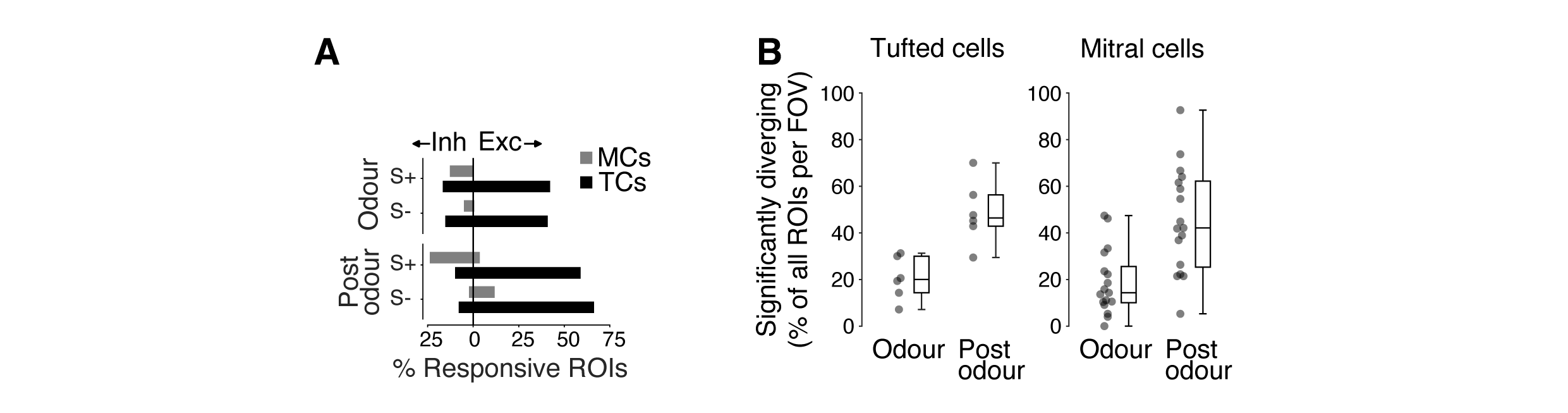

Supplement: S3 Fig — (A) Proportion of ROIs that respond with significant inhibition (leftward bar) and excitation (rightward bar) for mitral cells (grey) and tufted cells (black). (B) Proportion of ROIs that show significant divergence in response between S+ and S- odours. Odour period is during the 1 s odour presentation, while post-odour period corresponds to 1–3 s after the odour onset (0–2 s after the odour offset), before the reward delivery. (TIF) [file pbio.3002536.s003.tif]

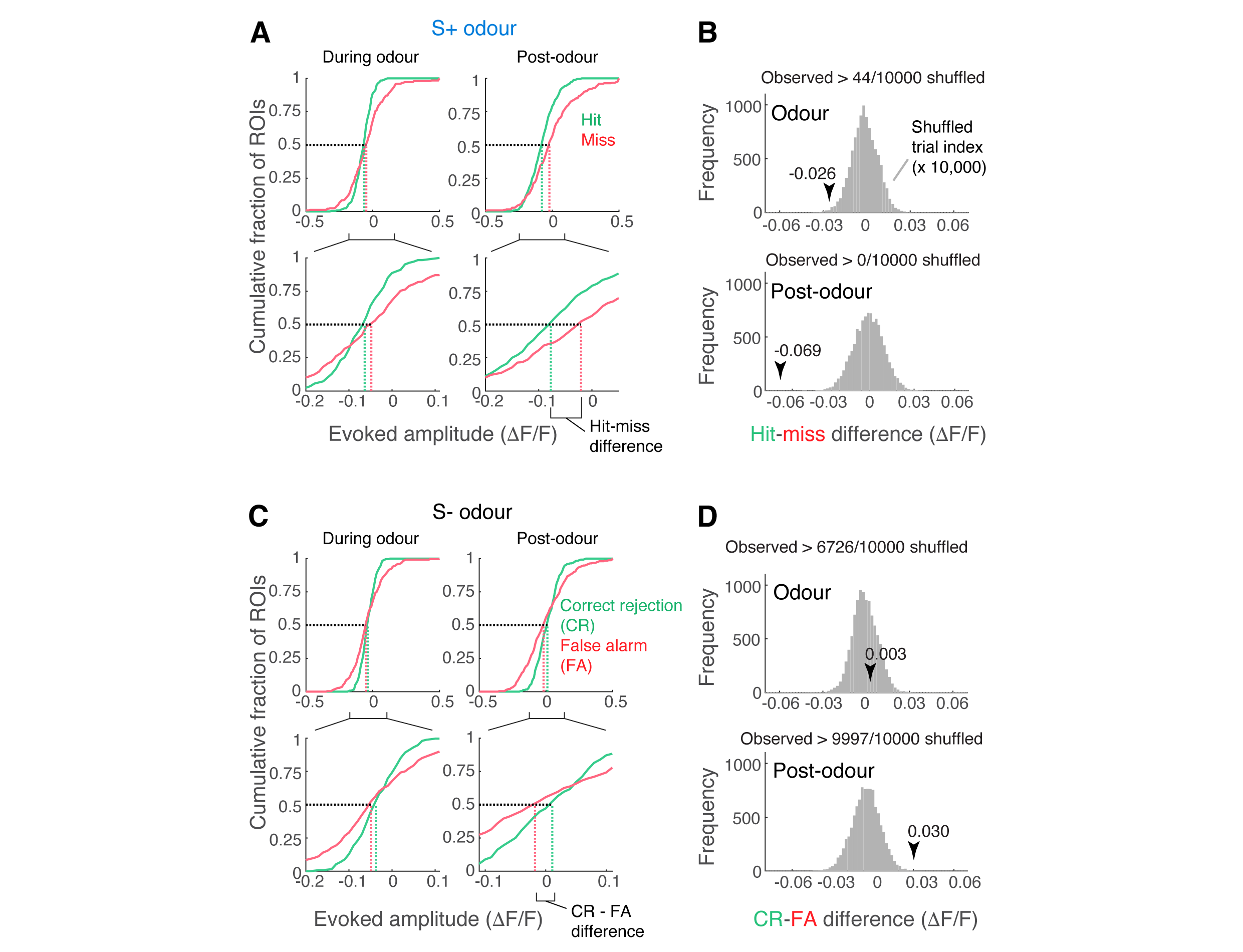

Supplement: S4 Fig — (A) Cumulative histograms of fluorescence changes during the odour (left) and post-odour (right), averaged over “Hit” trials (mice generate anticipatory licks in response to the S+ odour; green) vs. “Miss” trials (mice failed to generate anticipatory licks after the S+ odour presentation; red). Data is from mitral cell somata of Tbx21-Cre::Ai32 mice performing the difficult discrimination. Bottom row: same plots but with the x axis ranges indicated above. (B) The observed difference in the median evoked amplitude of the “Miss” distribution was subtracted from the median evoked amplitude from the “Hit” distribution in A (“Hit-miss difference”) and was compared against a shuffled distribution, where trial indices were randomly permutated. The random permutation was repeated 10,000 times. (C, D) Same as A, B, but for the S- odour, and correct and incorrect outcomes correspond to “Correct rejection” and “False alarm,” respectively. (TIF) [file pbio.3002536.s004.tif]

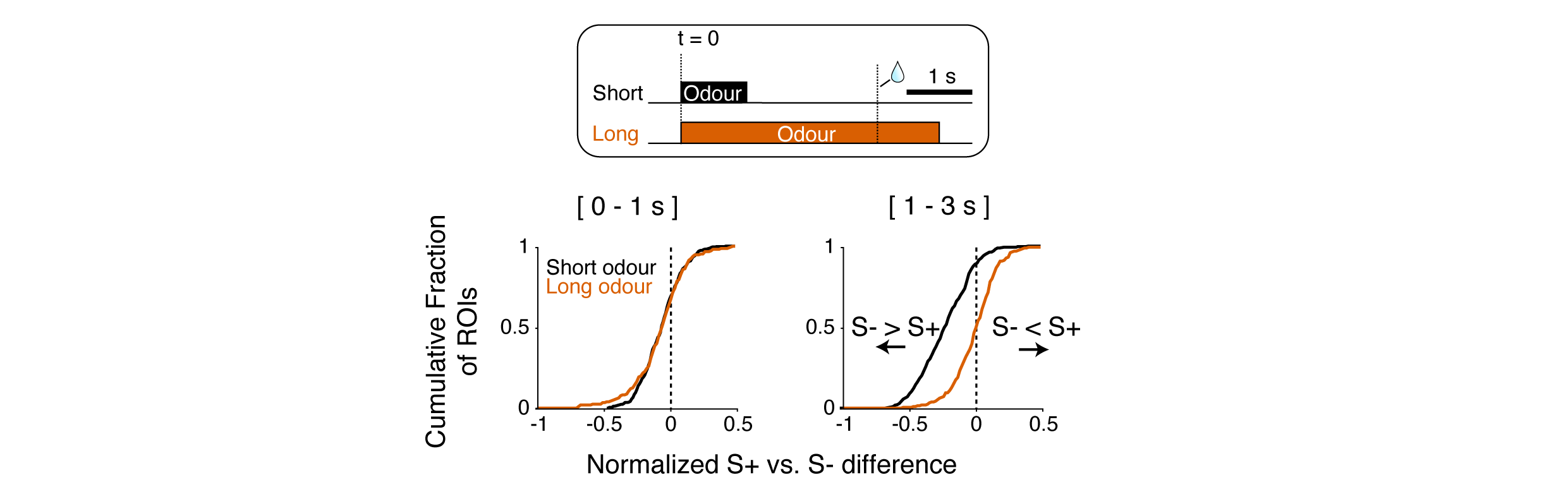

Supplement: S5 Fig — (A) Schematic showing the time course of short vs. long odour presentations. Short odour presentation involved 1-s presentation of odours, followed by a 2-s trace period before the reward delivery. With the long odour presentation, a 4-s odour presentation overlapped in time with the reward delivery, which occurred at 3 s. (B) Cumulative histograms of S+ vs. S- response amplitudes (normalised by the maximum magnitude for the entire dataset) for the short odour (black) and long odour (orange) experiments. (TIF) [file pbio.3002536.s005.tif]

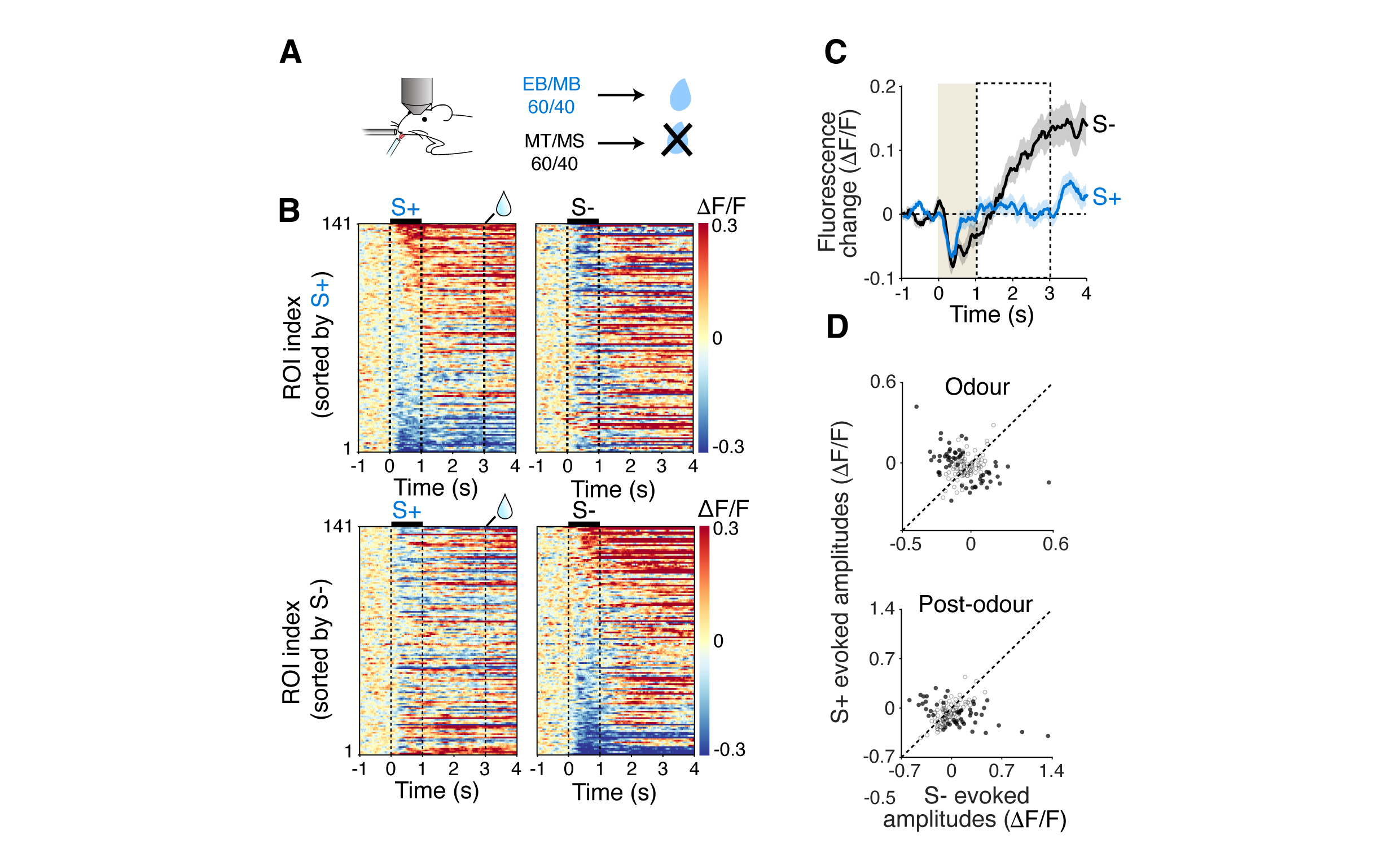

Supplement: S6 Fig — (A) Schematic of the easy discrimination task. (B) Colormap representation of S+ and S- responses imaged from mitral cell somata in Lbhd2-CreERT2::Ai95D mice performing the easy task. Top, ROI indices were sorted by the S+ response amplitudes; bottom, ROI indices were sorted by the S- response amplitudes. (C) Average fluorescence change of all ROIs (mitral cell somata) for the S+ (blue) and S- (black) odours. (D) Scatter plot comparing S- vs. S+ responses for odour (top) and post-odour (bottom) periods. Each point represents 1 ROI, and the data shown are from all sessions and mice. Dotted line represents unity (S- amplitude = S+ amplitude). Individual points correspond to ROIs. Black dots indicate S+ and S- responses that were significantly different. (TIF) [file pbio.3002536.s006.tif]

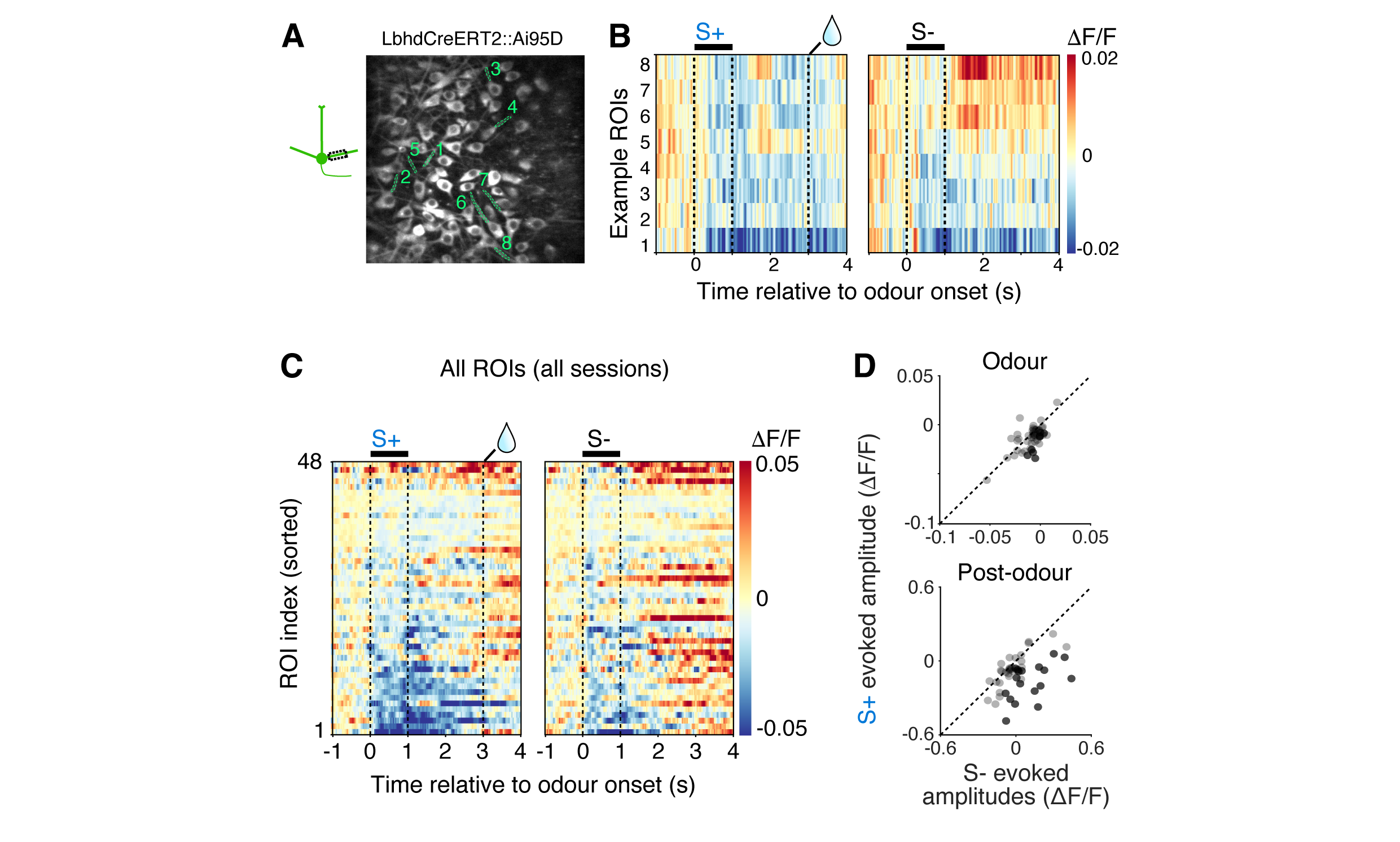

Supplement: S7 Fig — (A) An example field of view from an Lbhd2-CreERT2::Ai95D mouse at the mitral cell layer. ROIs were manually drawn around the lateral dendrites proximal to the somata as illustrated in the schematic (left). (B) Normalised fluorescence change (ΔF/F) for the ROIs indicated in A around the time of the rewarded odour (left) and unrewarded odour (right). (C) Colormap representation of normalised fluorescence change (ΔF/F) for all ROIs. (D) Scatter plots comparing the amplitude of fluorescence change evoked by S+ odour vs. S- odour for the odour period (above) and post-odour period (below). Dots correspond to ROIs from all animals shown. Black dots indicate significantly divergent responses. (TIF) [file pbio.3002536.s007.tif]

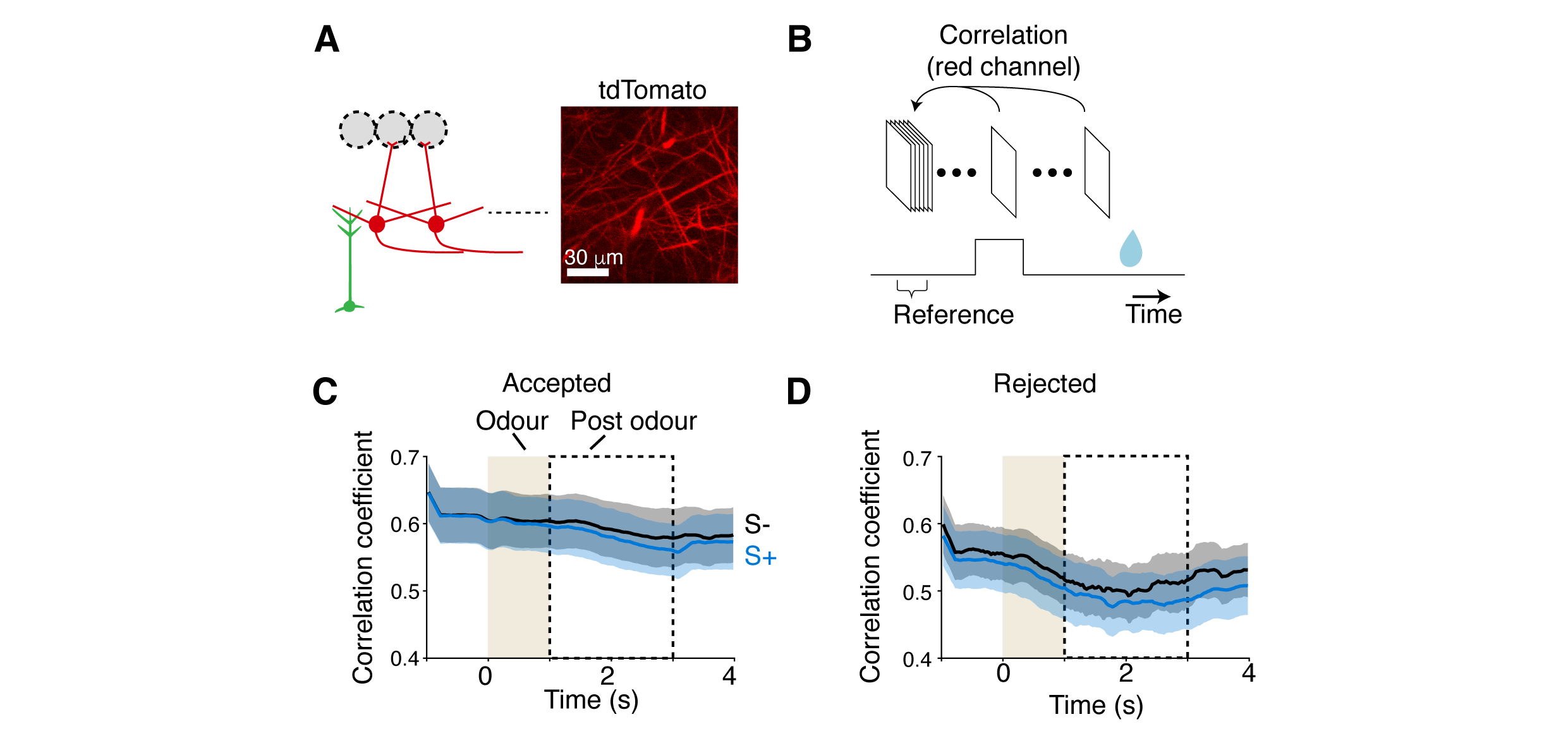

Supplement: S8 Fig — (A) Example field of view from the red channel showing mitral cell dendrites. (B) Image quality was determined by the frame-by-frame similarity of red fluorescence patterns by calculating correlation in the tdTomato image between the baseline period and other time points within the trial. Those with a high correlation coefficient throughout the trial is deemed to have less drift, e.g., due to animal’s movements. (C) Time course of red fluorescence correlation values for the accepted dataset. (D) Same as C but for the rejected dataset. Of the 1,917 trials imaged in total 574 trials were accepted and 1,343 trials were rejected. This amounts to, on average, 27.7% acceptance rate for deep gemmules and 36.2% acceptance rate for superficial gemmules. (TIF) [file pbio.3002536.s008.tif]
